# Chemical mixtures and fluorescence in situ hybridization analysis of natural microbial community in the Tiber river

**DOI:** 10.1016/j.scitotenv.2019.04.011

**Published:** 2019-07-10

**Authors:** Maria Ludovica Saccà, Valentina Elisabetta Viviana Ferrero, Robert Loos, Martina Di Lenola, Simona Tavazzi, Paola Grenni, Nicoletta Ademollo, Luisa Patrolecco, Jim Huggett, Anna Barra Caracciolo, Teresa Lettieri

**Affiliations:** a National Research Council, Water Research Institute, Via Salaria km 29,300, 00015 9 Monterotondo, Rome, Italy; b Molecular and Cell Biology team, LGC, Queens Road, Teddington, Middlesex TW11 0LY, United Kingdom; c European Commission, Joint Research Centre (JRC), Ispra, Italy; d School of Biosciences & Medicine, Faculty of Health & Medical Science, University of Surrey, Guildford, Surrey GU2 7XH, United Kingdom

**Keywords:** DAPI, 4′,6-diamidino-2-phenylindole, DOC, Dissolved Organic Carbon, EQS, environmental quality standards, FISH, Fluorescent In Situ Hybridization, LOQ, Limit of Quantification, PAH, Polycyclic Aromatic Hydrocarbon, PCA, Principal Components Analysis, PFAS, perfluoroalkyl substances, UBA, German on-line Information System on Ecotoxicology and Environmental Quality Targets, WFD, Water Framework Directive 2000/60/EC, Freshwater, Anthropogenic pollution and stressors, Bioindicators, Microbial populations, Water quality

## Abstract

The Water Framework Directive (WFD) regulates freshwater and coastal water quality assessment in Europe. Chemical and ecological water quality status is based on measurements of chemical pollutants in water and biota together with other indicators such as temperature, nutrients, species compositions (phytoplankton, microalgae, benthos and fish) and hydromorphological conditions. However, in the current strategy a link between the chemical and the ecological status is missing. In the present WFD, no microbiological indicators are foreseen for integrating the different anthropogenic pressures, including mixtures of chemicals, nutrients and temperature changes, to provide a holistic view of the freshwater ecosystem water quality. The main aim of this work was to evaluate if natural microbial populations can be valuable indicators of multiple stressors (e.g. chemical pollutants, temperature, nutrients etc.) to guide preventive and remediation actions by water authorities. A preliminary survey was conducted to identify four sites reflecting a contamination gradient from the source to the mouth of a river suitable to the objectives of the European Marie Curie project, MicroCoKit. The River Tiber (Italy) was selected as a pilot case study to investigate the correlation between bacteria taxa and the chemical status of the river. The main physicochemical parameters, inorganic elements, organic pollutants and natural microbial community composition were assessed at four selected sites corresponding to pristine, agricultural, industrial and urban areas for three consecutive years.

The overall chemical results indicated a correspondence between different groups of contaminants and the main contamination sources at the selected sampling points. Phylogenetic analysis of the microbial community analyzed by Fluorescence In Situ Hybridization method (FISH) revealed differences among the four sampling sites which could reflect an adaptive bacterial response to the different anthropogenic pressures.

## Highlights

• Four freshwater sites differently affected by anthropogenic pressure were analyzed.
• The most chemical polluted site was the urban area.
• The bacterial community varies with degree of anthropogenic pressure.
• β-*Proteobacteria* were predominant in the urban area.

## Graphical abstract

**Figure:**
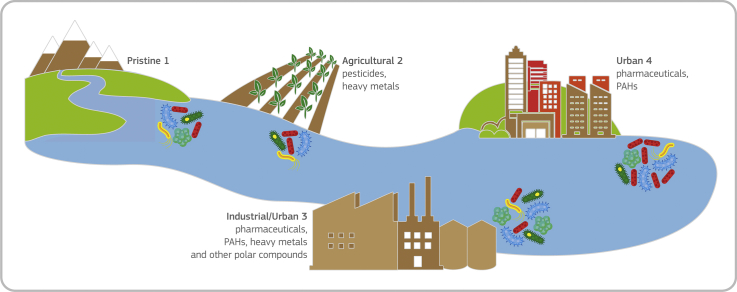

## 1 Introduction

In Europe many different chemical pollutants are released into the aquatic environment, mainly from agriculture, industry, and households. According to the European Water Framework Directive (WFD 2000/60/EC) (EU, 2000), a strategy for water protection that includes specific measures for pollution control to achieve a good ecological and chemical status at the European level has been established. The ecological quality assessments of freshwater bodies are based on biological quality elements (aquatic flora, benthic invertebrates and fish), hydromorphological parameters (depth, width, quantity and dynamics of water flow), physicochemical quality elements (temperature, oxygen balance, salinity, pH and nutrients) and the concentration of river basin chemical pollutants. The chemical status is evaluated by compliance with the established environmental quality standards (EQS) limit values for priority substances laid down in Directive 2008/105/EC and amended by Directive 2013/39/EU.

The implementation of the WFD has been addressed in the literature (Brack et al., 2017; Hering et al., 2010; Könemann et al., 2018; Reyjol et al., 2014; Vlachopoulou et al., 2014; Voulvoulis et al., 2017), and the need of integration of chemical and biological analyses has been pointed out (Geiszinger et al., 2009; Ricciardi et al., 2009). Estimation of the relationship of human pressures with the ecological status of European rivers has been recently carried out by considering indicators of pollution, hydrological and hydromorphological alterations (Grizzetti et al., 2017). Nevertheless, there are currently no microbiological indicators able to respond to multiple stressors and integrate different anthropogenic pressures, such as chemical mixtures, nutrients and temperature changes. Moreover, environmental samples are usually very complex and can contain numerous natural and anthropogenic chemicals, even though most are present in very low concentrations (Artigas et al., 2012). Microbial communities are the base of the food web pyramid, representing about 50% of the total biomass on Earth. They play a key role in ecosystem functioning and in several ecosystem services by being responsible for geochemical cycles and bio-removal of organic compounds, including xenobiotics (Turbé et al., 2010). Traditionally, pathogen microorganisms have been used as indicators of fecal contamination, however natural microbial populations can be used to assess the functional efficiency of ecosystems being useful for environmental assessment as complementary methods to chemical monitoring (Boi et al., 2016). For these reasons, the changes that can be observed in a microbial community can be analyzed from a global and multivariate perspective, to understand and assess the impact of the wide and complex mixture of chemical and physical parameters on the ecological status of the environment.

An environmental indicator should reflect environmental changes in a robust way, be capable of being monitored with relative ease, be cost effective and provide an early warning of the potential effects of a stressor in the environment. Two main approaches can be used to study microbial groups associated with contamination, one is based on the identification of taxa able to resist and/or to degrade chemicals, and the other on the detection of marker genes independently from their taxonomic group (Fields et al., 2006; Martín et al., 2008; Lammel et al., 2015). The identification of biological indicators capable of responding to different stressors can be very useful for providing a global view of the freshwater ecosystem and therefore water quality, not exclusively linked to individual quality elements such as the different priority substances but to the complex mixture of chemical and physical parameters (Artigas et al., 2012). In this context, the aim of this work was the identification of microbial community-based indicators for monitoring and evaluating the complexity of multiple stressors relevant to guiding preventive and remediation actions by water authorities. Chemical, physicochemical, and biological analyses were performed at four sampling sites along the course of the River Tiber (Italy), reflecting different anthropogenic pressures to assess contaminations and possible relationships between the chemical pollutants, physicochemical parameters, nutrients and the microbial community. In this study the microbial community was analyzed by FISH, a technique which permitted to identify, without extracting nucleic acids, active microbial cells at different phylogenetic levels under an epifluorescence microscope. FISH combines the precision of molecular genetics with the direct visual information from microscopy, allowing simultaneous visualization, identification, enumeration and localization of individual microbial cells within their natural microhabitat (Di Lenola et al., 2017).

## 2 Materials and methods

Chemical reagents for analytical methods such as HPLC grade acetonitrile, methanol, hexane and acetone were from Merck (Darmstadt, Germany). HPLC grade methylene chloride came from Sigma Aldrich (Oakville, ON, USA). Polycyclic Aromatic Hydrocarbon (PAHs) were purchased as stock solution of 100 mg/L in cyclohexane from Aldrich (Steinheim, Germany) at 98% purity.

### 2.1 Study area and river sampling points

**Fig. 1:**
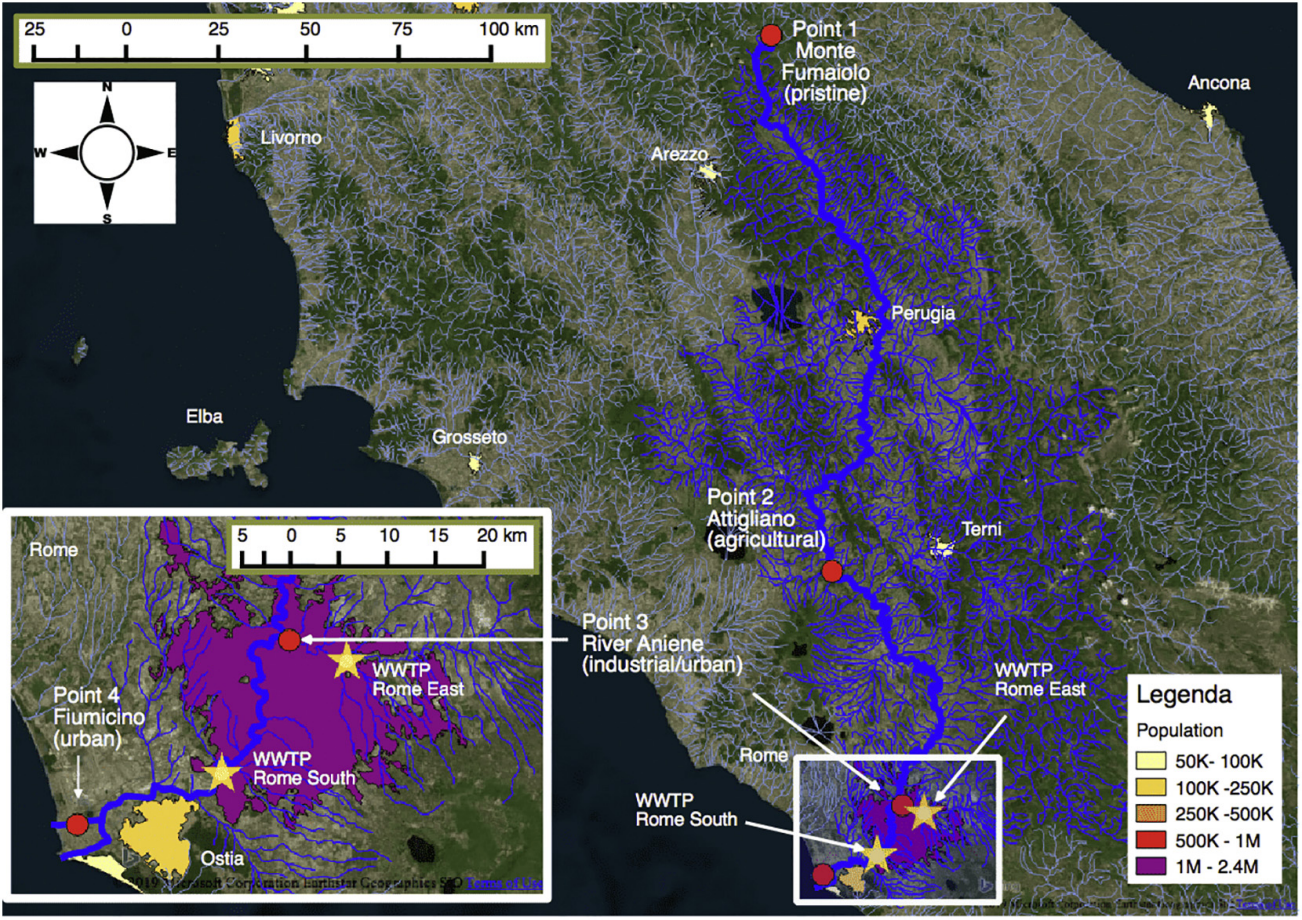
Geographic map of the four sampling points along the River Tiber (central Italy). The South and East wastewater treatment plants (WWTP) of Rome are indicated with the yellow stars.

The Tiber is the main river in central and peninsular Italy; it is the third longest (409 km) and the second largest (17,375-km^2^) river basin in the entire national territory. It rises in Emilia-Romagna (Monte Fumaiolo) and flows through the Tuscany, Umbria and Lazio regions. In Rome, it receives water from one of its main tributaries, the River Aniene, then flows through Rome and finally enters the Thyrrhenian Sea in Fiumicino and Ostia. The total human population in the River Tiber basin is approximately 4.7 million. The annual mean flow in the lowest part of the river is 260 m^3^/s (Fig. 1).

The four selected sampling points comprised one pristine reference site and three other points characterized by different levels or type of pollution. Point 1: pristine area, Pieve di Santo Stefano (43°42′26″N, 12°01′50″E) (only in the first sampling campaign conducted in autumn October 2013), afterwards changed to Monte Fumaiolo (43°47′11″N, 12°04′50″E) at the source of the river, due to unexpected contamination of the first point (see paragraph 3.6). Point 2: agricultural area, Attigliano (in the Province of Terni, Umbria region, 42°30′30″N, 12°16′59″E), an area possibly affected by widespread pesticide use. Point 3: industrial and urban area, where River Aniene flows into the River Tiber in Rome (41°56′22″N, 12°30′26″E), downstream from Rome East wastewater treatment plant (WWTP). Point 4: urban area, Fiumicino, downstream from the Southern WWTP of Rome and close to the river mouth (41°48′15″N, 12°14′50″E) (Fig. 1 and paragraph 1 in SI).

### 2.2 Sample collection and processing

The water samples (0–20 cm from the surface) were collected in triplicates at each of the four sampling points in the five sampling campaigns comprising consecutive seasons of autumn and spring (October 2013, March 2014, October 2014, April 2015 and October 2015). At point 1 (Monte Fumaiolo) the Tiber is a very small mountain stream. At points 2 and 3, sampling was performed in the middle of the river from a bridge by lowering a multi-parametric probe and a bucket. Sampling at point 4 was only possible from the riverbank. The main physicochemical parameters such as temperature, pH, redox potential (mV), conductivity (μS/cm), and dissolved oxygen (mg/L) (or oxygen saturation in %) (Table SI1) were measured during sampling (on-site) using a Hydrolab DSS multi-parameter water quality probe (Ott Hydromet; Kempten, Germany). All water samples were filtered through a 0.5 mm steel sieve to remove any coarse particles. Samples for analyses of metals and Dissolved Organic Carbon (DOC) were collected in polyethylene bottles previously washed with HNO_3_ (pH < 2) for at least 24 h and then washed with milliQ water until a neutral pH was reached.

Water samples for microbiological analysis (cell viability, cell abundance and Fluorescent In Situ Hybridization: FISH analysis) were collected using polyethylene sterile bottles (3 bottles of 1 L each), and were processed immediately after sampling. For organic contaminants and polycyclic aromatic hydrocarbons (PAHs), water samples were transported and stored in pre-cleaned 2.5 L glass bottles (3 replicates) and stored at 4 °C until analysis was performed. Triplicate water samples for the analysis of the polar organic pollutants analyzed by UHPLC-MS-MS were collected and stored in 1 L aluminum bottles (Scientifica Panzeri, Milan, Italy) at 4 °C. In addition, blank control samples were taken at each sampling point by simply opening the aluminum bottle during sampling.

### 2.3 Chemical analysis of inorganic ions, metals and DOC

Analyses of inorganic ions and metals were performed in accordance with Italian Official Guideline (APAT-IRSA, 2003) and APHA, AWWA, WEF (1995) methods. To ensure stability of the chemicals prior analysis, samples were filtered immediately by gentle vacuum through 0.45 μm polycarbonate filters. Inorganic anions (fluoride, chloride, nitrite, nitrate and sulphate) were determined by ion chromatography using a Dionex DX-120 Ion Chromatograph. A pre-acidification step (1% HNO_3_) was performed for metal and cation analyses. Metals (barium, antimony, arsenic, cadmium, total chromium, copper, lead, mercury, nickel, selenium, vanadium, iron, zinc, manganese, aluminum, lithium, cesium, uranium, cobalt, strontium) were analyzed by Inductively Coupled Plasma-Mass Spectrometry (ICP-MS) (Agilent technologies 7500c), with Octopole Reaction System (ORS) (Table SI2). Major cations (calcium, magnesium, sodium, potassium) were determined by an Inductively Coupled Plasma Optical Emission Spectroscopy (ICP-OES) using a Perkin Elmer P400 spectrometer (Table SI3). The Dissolved Organic Carbon (DOC) was determined in pre-filtered water samples (GF/F glass fiber filters, pre-combusted at 450 °C for 4 h) by High Temperature Catalytic Oxidation (HTCO), using the Shimadzu TOC-5000 analyser, with a detection limit of 0.2 mg/L (Table SI3).

Detection limits (LODs) were 0.01 μg/L for all the metals except for Sr (0.1 μg/L). LODs were calculated, in accordance with the IUPAC Method (1999), from the lowest analyte concentration producing a peak that could be reliably distinguished from the noise (three-time signal to noise ratio). The quantification limits (LOQs) were set at 3 times the LODs.

### 2.4 PAHs analysis

Naphthalene, acenaphthene, fluorene, phenanthrene, anthracene, fluoranthene, pyrene, benzo(a)anthracene, chrysene, benzo(b)fluoranthene, benzo(k)fluoranthene, benzo(a)pyrene, dibenzo(a,h)anthracene, benzo(g,h,i)perylene, and indeno(1,2,3-c,d)pyrene standard solutions (1 mg/L) were prepared by dilution of each stock solution with acetone and stored at 4 °C.

Methanol in a ratio 1:200 (v/v) was added to 1–2 L of filtered water sample and it was then extracted by solid-phase extraction (SPE), following the method reported in Patrolecco et al. (2010). Analytical determination of PAHs was performed by a RP-HPLC (Varian 9012) coupled to a fluorescence detector (Perkin Elmer LS4). The detection limits were in the range of 0.01–0.5 ng/L for all PAHs in the water samples (Table SI4).

### 2.5 Polar organic contaminants

Rationale for the selection of these contaminants is explained in Supplementary information (SI paragraph 2).

Organic contaminants were extracted from 1 L water using automated solid-phase extraction (SPE) with Oasis HLB (200 mg) cartridges (Waters Corporation, Milford, MA, USA) using an Autotrace AT280 SPE workstation (Thermo Scientific, Waltham, MA, USA). Analyses were performed by ultra-high pressure liquid chromatography tandem mass spectrometry (UHPLC-MS-MS), which was performed with an Acquity® UHPLC system (Waters) coupled to a hybrid triple-quadrupole linear ion trap mass spectrometer 5500 QTRAP® with a turbo ion spray source from AB SCIEX (Foster City, CA, USA). Experimental details are given in Loos et al. (2017). The internal surrogate standards used for “isotope dilution” quantification are depicted in Table SI5. The Limits of Quantification (LOQs) for the target analytes are shown in Table SI6.

### 2.6 Environmental quality standards (EQS)

The EQS were selected from Directive 2013/39/EU (EU, 2013), the Swiss Ecotox Centre (http://www.ecotoxcentre.ch/expert-service/quality-standards), the German on-line Information System on Ecotoxicology and Environmental Quality Targets (UBA) (https://webetox.uba.de/webETOX/public/search/ziel.do), and for the perfluoroalkyl substances from the substance dossiers of the Italian EQS working group (Valsecchi et al., 2017).

### 2.7 Analysis of microbiological parameters

Water samples for microbial abundance and phylogenetic characterization were fixed with formaldehyde (2% final concentration) and volumes ranging from 0.5 to 3 mL were filtered through a 0.2 μm polycarbonate membrane (Merck Millipore) by gentle vacuum (<0.2 bar). The volume of filtered water was adjusted for each sample in order to obtain enough cells for a representative enumeration.

Microbial abundance was assessed immediately after filter preparation, whereas filters for phylogenetic characterization were stored at −20 °C until processing.

Filters for microbial abundance evaluation by total direct count (cells/mL) were treated using DAPI (4′,6-diamidino-2-phenylindole) as the nucleic acid stain (1 μg/mL). Cells were visualized and enumerated with a fluorescence microscope (Leica DM 4000B, Leica Microsystems GmbH, Wetzlar, Germany). A minimum of 300 cells were counted for each replicate in at least 30 fields selected randomly on each filter (Barra Caracciolo et al., 2010).

The percentages of live cells were measured in non-fixed fresh samples filtered as described above, and stained with two fluorescent dyes, SYBR Green II and propidium iodide (Sigma–Aldrich, Germany) (Grenni et al., 2014).

The bacterioplankton phylogenetic composition was analyzed by the Fluorescence In Situ Hybridization method (FISH), using Cy3-labelled oligonucleotide probes (Biomers.net, Ulm, Germany) targeting the dominant bacterial taxa found in freshwater ecosystems (Pernthaler, 2013) (Table 1). This method made it possible to assess bacterial diversity at phylum and class level. The cells binding each probe were estimated as a proportion of the total DAPI-positive cells (% positive cells vs DAPI). FISH analysis was performed according to previously published protocols (Barra Caracciolo et al., 2010; Grenni et al., 2014; Patrolecco et al., 2018). Each microbiological analysis was performed in three replicates for each sampling point.

**Table 1:** List of the rRNA-targeted probes used for Fluorescence in Situ Hybridization (FISH) analysis, corresponding microbial taxa identified, oligonucleotide sequence and rRNA position. Further information is available at http://www.microbial-ecology.net/probebase (Greuter et al., 2016).

Target taxa	Probe	Sequence (5′-3′)	rRNA position
*Archaea*	ARCH915	GTGCTCCCCCGCCAATTCCT	16S(915 – 934)
*Bacteria*	EUB338 EUB338II EUB338III	GCTGCCTCCCGTAGGAGT GCAGCCACCCGTAGGTGT GCTGCCACCCGTAGGTGT	16S(338-355)
*α-Proteobacteria*	ALF1b	CGTTCGYTCTGAGCCAG	16S(19-35)
*β-Proteobacteria*	BET42a	GCCTTCCCACTTCGTTT	23S(1027-1043)
*γ-Proteobacteria*	GAM42a	GCCTTCCCACATCGTTT	23S(1027-1043)
*δ-Proteobacteria*	DELTA495a DELTA495b DELTA495c	AGTTAGCCGGTGCTTCCT AGTTAGCCGGCGCTTCCT AATTAGCCGGTGCTTCCT	16S(495-512)
*ε-Proteobacteria*	EPS710	CAGTATCATCCCAGCAGA	16S(710-727)
*Planctomycetes*	PLA46 PLA886	GACTTGCATGCCTAATCC GCCTTGCGACCATACTCCC	16S(46-63)
*Bacteroidetes* (CF)	CF319a	TGGTCCGTGTCTCAGTAC	16S(319-336)
*Firmicutes*	LGC354a LGC354b LGC354c	TGGAAGATTCCCTACTGC CGGAAGATTCCCTACTGC CCGAAGATTCCCTACTGC	16S(354-371)
*Actinobacteria*	HGC69a	TATAGTTACCACCGCCGT	23S(1901-1918)

### 2.8 Fecal contamination

The fecal contamination was assessed for a preliminary microbiological analysis of the sampling sites by analysing total coliforms, *E. coli* and *Enterococcus* spp. using the commercial fecal indicator assays Colilert and Enterolert (IDEXX Laboratories, Westbrook, ME). The values are reported as the most probable number (MPN)/100 mL.

### 2.9 Data analysis

The data obtained from the upstream point Pieve di Santo Stefano in the first sampling campaign (autumn 2013) were excluded from data analysis due to unexpected microbial and chemical contamination (see paragraph 3.6). The values < LOQ were set as half of the LOQ for average calculation (according to Directive 2009/90/EC). Statistical analyses were performed using the software PAST, version 3.11 (Hammer et al., 2001). All analyzed parameters were grouped for data analysis as described in Table 2 Analysis of variance was performed with log transformed data considering sampling campaigns, sampling points and seasons as variance factors. The correlation between microbiological parameters and grouped chemical compounds was assessed by the Spearman's non-parametric rank-order correlation coefficient by including all five samplings and evaluating sites separately. The Bonferroni correction for multiple testing was incorporated by setting the significance cut-off by dividing the P value for the number of tests to be conducted. The Principal Components Analysis (PCA) between sampling points was performed on a correlation matrix, implying standardization of variables, calculated using normalized data values from the grouped chemical compounds or from the microbial phylogenetic structure using division by their standard deviations (Ramette, 2007). SIMPER (Similarity Percentage) was performed to assess which taxa were primarily responsible for the observed differences between samples using Bray Curtis distance similarity. The bacterial diversity in each point was estimated by calculating Shannon and Simpson diversity indices using the bacterial taxa abundance data set (cells/mL).

**Table 2:** List of parameters analyzed in the River Tiber and grouped as described in the table: physicochemical parameters, PAHs, polar organic contaminants (including benzotriazoles, pharmaceuticals, pesticides, perfluorinated compounds), microbiological parameters, ions and nutrients, metals.

Physicochemical parameters	PAHs	Polar organic contaminants	Microbiological parameters	Ions and nutrients	Metals
T (°C)	Naphtalene	**Benzotriazoles**	Cell Viability	NO_2_^−^	Ba
pH	Acenaphtene	1H-Benzotriazole	Microbial abundance	NO_3_^−^	Sb
Redox potential (mV)	Fluorene	Methylbenzotriazoles	*Bacteria*	Cl^−^	As
Conductivity (μS/cm)	Phenanthrene	**Pharmaceuticals**	*Archaea*	SO_4_^2−^	Cd
Dissolved oxygen saturation (%)	Anthracene	Carbamazepine (CBZ)	*α-Proteobacteria*	F^−^	Cr
Dissolved oxygen (mg/L)	Fluoranthene	CBZ metabolite	*β-Proteobacteria*	Ca^2+^	Cu
	Pyrene	Diclofenac	*γ-Proteobacteria*	Mg^2+^	Pb
	Benzo(a)anthracene	Sulfamethoxazole	*δ-Proteobacteria*	Na^+^	Hg
	Chrysene	**Pesticides**	*ε-Proteobacteria*	K^+^	Ni
	Benzo(b)fluoranthene	2,4-D	*Planctomycetes*	B^+^	Se
	Benzo(k)fluoranthene	DEET	*Bacteroidetes*	DOC	V
	Benzo(a)pyrene	Cybutryne (Irgarol)			Fe
	Dibenz(ah)anthracene	MCPA			Zn
	Benzo(g,h,i)perylene	Metolachlor			Mn
	Indeno(1,2,3 cd)pyrene	Terbutryn			Al
		Terbutylazine			Li
		**Perfluorinated compounds**			Cs
		PFBS			U
		PFHxA			Co
		PFHpA			Sr
		PFOA			
		PFNA			
		PFOS			

## 3 Results

The complete list of all parameters analyzed, and how they were grouped for data analysis, at the four sampling points is shown in Table 2.

### 3.1 Physicochemical parameters

**Fig. 2:**
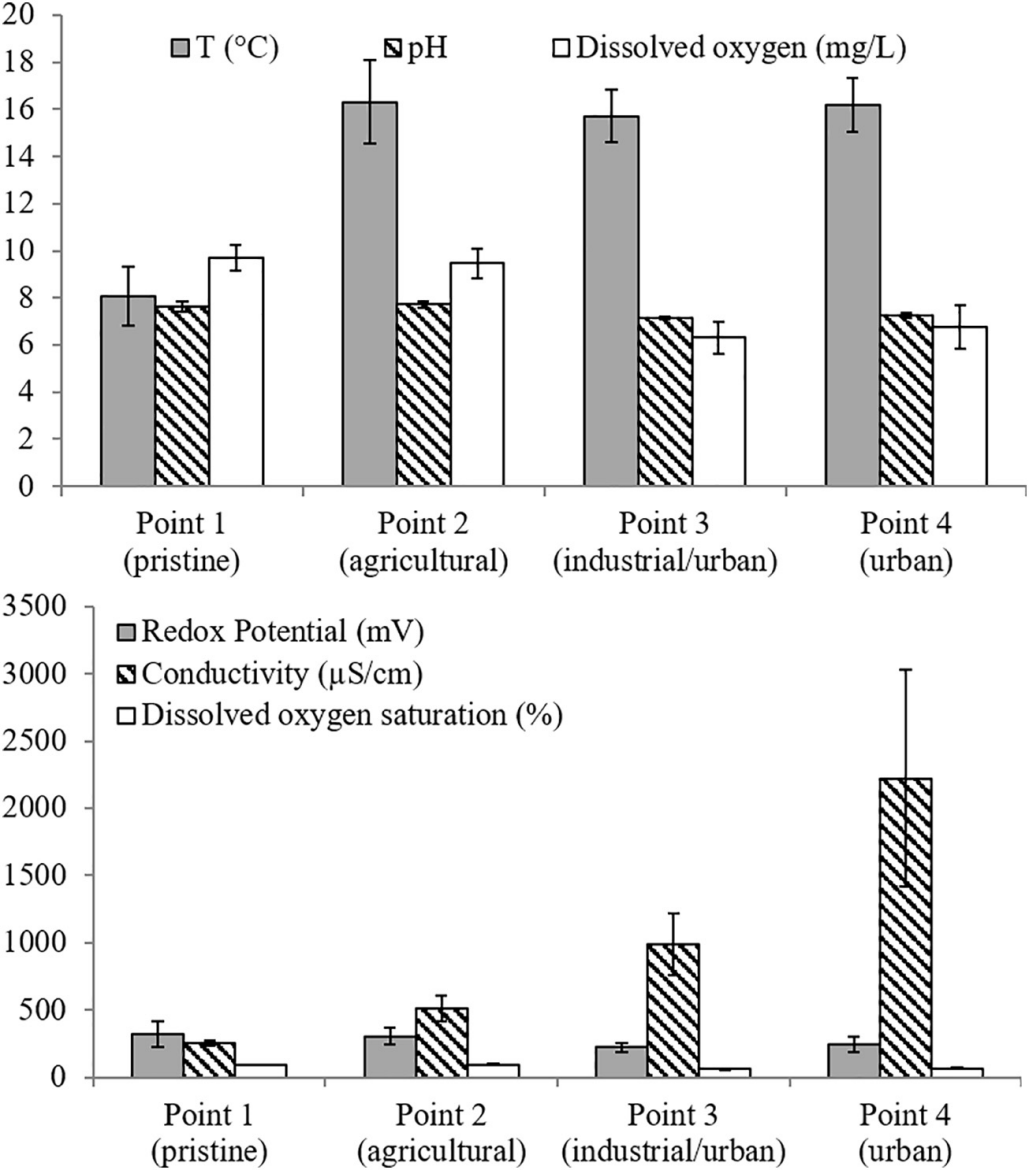
Main physicochemical parameters. Average values of the five samplings for each selected point (1, 2, 3 and 4) of the river. The vertical bars represent the standard errors.

The results of the physicochemical parameters (T, pH, redox potential, conductivity, dissolved oxygen) measured at the four sampling points are given in Table SI1. As expected the average temperature was lower at the pristine site (8.1 °C) compared to the other three points (16.1 °C). The pH was slightly higher at points 1 and 2 (pH 7.7 in both) compared to the industrial/urban points 3 and 4 (pH 7.2 and 7.3, respectively). The redox potential was similar at all four points. Higher differences in conductivity and dissolved oxygen concentration were observed among the points, remarkable was the conductivity increase and dissolved oxygen decrease from the pristine point to the river mouth (point 4). In particular, a ten-fold (average) increase in conductivity was observed from 257 to 2223 μS/cm (Fig. 2).

### 3.2 Inorganic ions and Dissolved Organic Carbon (DOC)

**Fig. 3:**
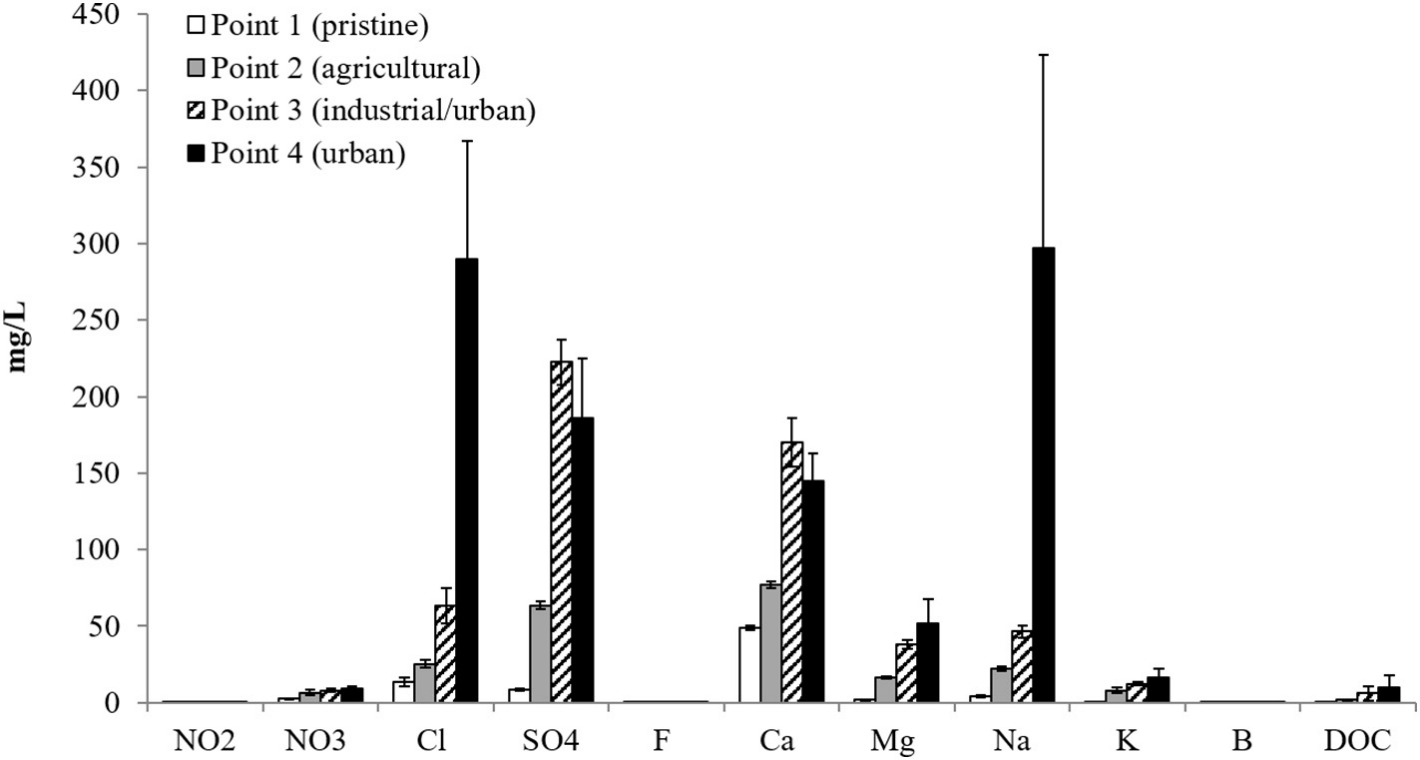
Inorganic ions and Dissolved Organic Carbon (DOC). Average concentrations (mg/L) of the five samplings for each selected point (1, 2, 3 and 4) of the River Tiber. The vertical bars represent the standard of the mean.

A general increasing trend in the anions and cations was observed from the river source to the mouth (Fig. 3 and Table SI3). At the pristine point 1, the DOC and all ion concentrations except calcium (average 49 mg/L) were very low (Fig. 3). The highest nitrate values were observed at point 4 (urban site; average 9.3 mg/L), except for two temporal peaks at the agricultural site (point 2) in the first two campaigns (October 2013 and March 2014, Table SI2). Nitrates ranged from 1.8 to 12.1 mg/L throughout the river course never exceeding the European limit value of 50 mg/L given in the Nitrates Directive (91/676/EC) and Groundwater Directive (2006/118/EC).

The increase in conductivity from the river source to the river mouth is ascribable to the increase in the main anion and cation values; in a similar way, the dissolved oxygen decrease was in accordance with the DOC increase throughout the river course. The chloride and sodium ion peaks found at point 4 (average 290 and 297 mg/L, respectively) can be explained by the saline sea intrusion in this area. At point 4 the highest temporal values for Cl^−^, SO_4_^2−^, Mg^2+^, Na^+^, K^+^, B^+^, and DOC were observed in October 2015 due to the high river flow (Table SI2).

### 3.3 Metals

**Fig. 4:**
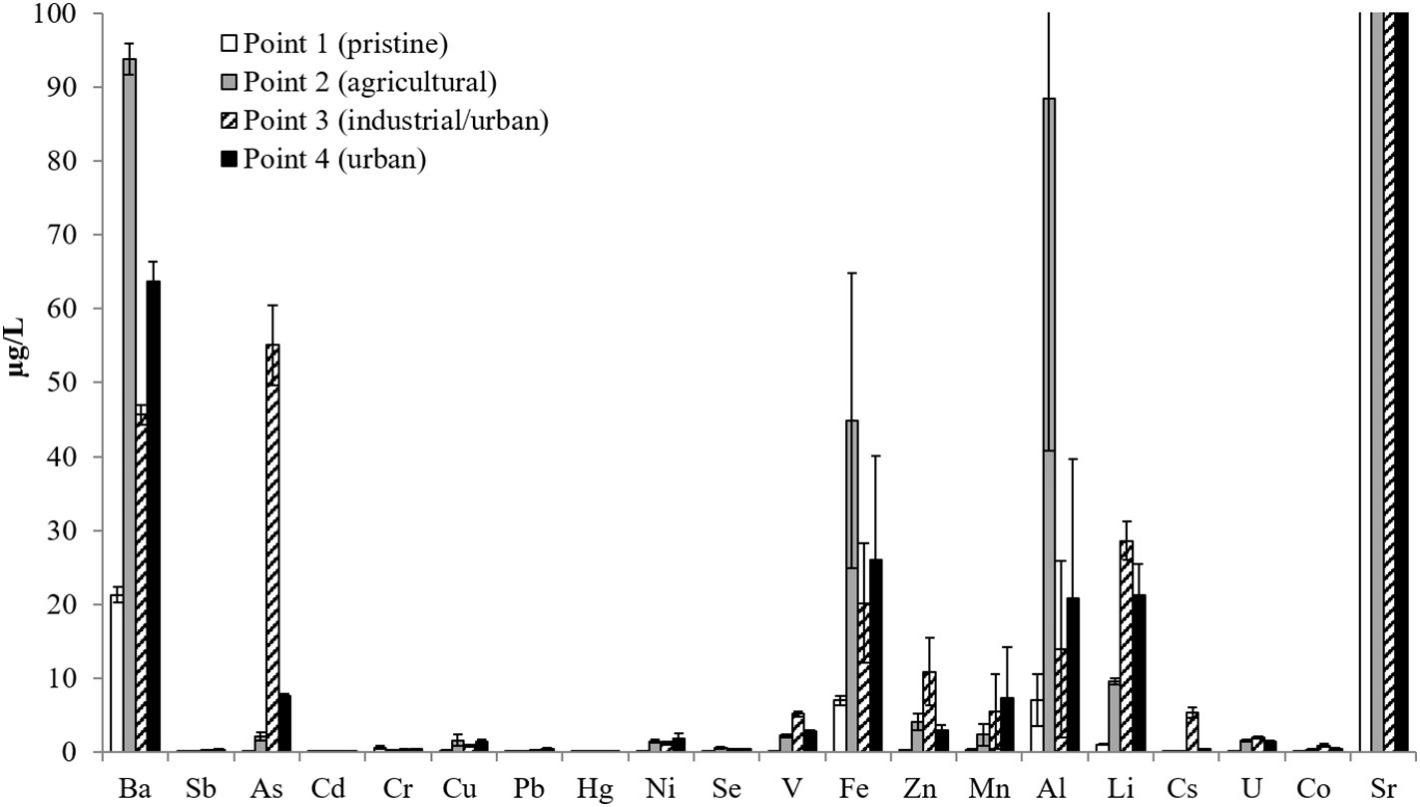
Average concentrations of the metals (μg/L) measured in the five samplings at the four sampling points (1, 2, 3 and 4) of the river. Out of scale values of Sr are indicated on the right (Point 1: 304 μg/L; Point 2: 800 μg/L; Point 3: 2138 μg/L; Point 4: 1411 μg/L). The vertical bars represent the standard errors.

The overall highest values of metals were observed in the March 2014 campaign, and the highest values were found in the industrial point 3 (Fig. 4 and Table SI3). Concentrations of strontium (Sr) in the four points are out of range in Fig. 4 (respectively 304, 800, 2138 and 1411 μg/L). The average concentrations ranged from 304 μg/L at point 1 (pristine) to 2138 μg/L at point 3 (industrial and urban area).

At the sampling point 3, arsenic (As) exceeded (average value of 55 μg/L) the Italian legal limit of 10 μg/L in all sampling campaigns and vanadium (V) exceeded the EQS of 3.5 μg/L (ranging from 4.3 to 6.3 μg/L).

At the sampling point 2 (agricultural area), the highest barium (Ba) levels (average 94 μg/L) were found, exceeding the EQS of 72 μg/L in all cases; lower values were observed at the other sampling points. Moreover, peaks in aluminum (Al) concentrations of 44, 246 and 148 μg/L respectively, were observed in the first 3 campaigns, and exceeded the EQS of 40 μg/L (Fig. 4).

In the case of Zn, the EQS of 20.6 μg/L was exceeded only in one case (28.5 μg/L) at point 3 in the sampling of October 2015.

Overall, the values of the metals found in all sampling points are in the range of those found in central Italian rivers and except for As they did not exceed the current Italian legal limits (Legislative Decree No. 152/2006).

### 3.4 Polycyclic aromatic hydrocarbons (PAHs)

**Fig. 5:**
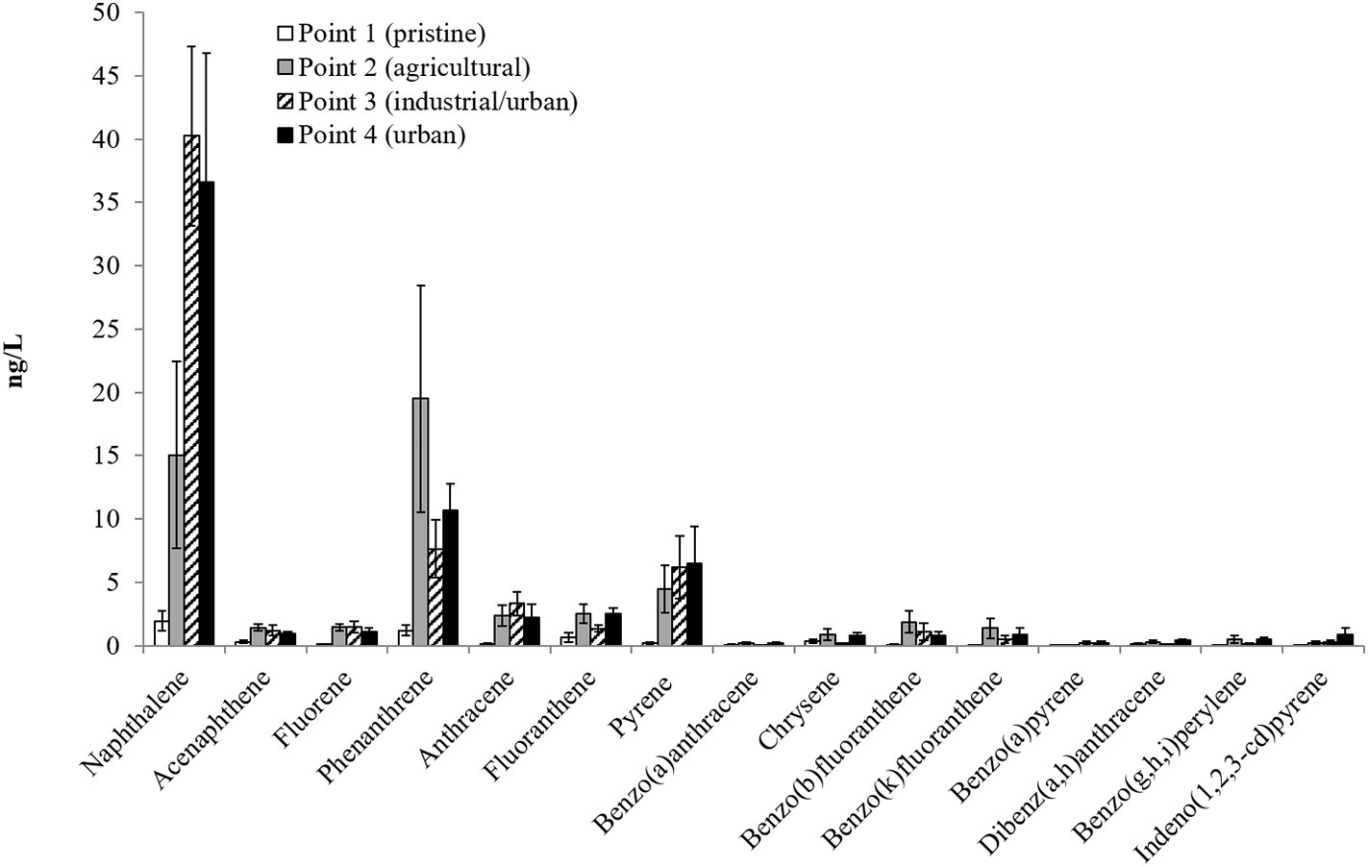
Average concentrations of the polycyclic aromatic hydrocarbons (PAHs) (ng/L) measured in the five samplings in the four selected points (1, 2, 3 and 4). The vertical bars represent the standard errors.

PAHs concentrations were generally low (Fig. 5 and Table SI4). The highest concentrations were found for naphthalene (max. 70 ng/L), phenanthrene (max. 44 ng/L), and pyrene (max. 15 ng/L) at points 2 and 4. At point 1 all concentrations were low, except in October 2013 when the sampling was performed in Pieve di Santo Stefano (about 20 km downstream from Monte Fumaiolo). The total PAHs concentrations detected in the dissolved water phase were 5 ng/L at point 1, 52 ng/L at point 2, 64 ng/L at point 3, and 65 ng/L at point 4.

Slight EQS exceedances were observed for benzo(a)anthracene (EQS: 0.23 ng/L) at point 2 in April and October 2015 (0.36 ng/L and 0.48 ng/L respectively) and at point 4 in October 2015 (0.37 ng/L), for chrysene (EQS. 1.2 ng/L) at point 2 in April and October 2015 (2.0 ng/L in both cases), and at point 4 in October 2015 (1.6 ng/L), and for indeno(1,2,3-cd)pyrene (EQS. 2.0 ng/L) at point 4 in October 2015 (2.8 ng/L). PAHs are wide ubiquitous contaminants due mainly to natural incomplete combustion processes and anthropogenic emissions and they can reach surface waters in different ways, including atmospheric deposition, urban run-off, municipal and industrial effluents (Montuori et al., 2016; Patrolecco et al., 2010). The two (naphtalene 44.2 ng/L) and three rings congeners (phenanthrene 44.0 ng/L) found at point 2 (in spring 2014) are directly related to petrogenic inputs, presumably caused by the proximity of a highway (A1 highway). Point 3 is located close to a motorway (Via Salaria) affected by an intense urban vehicle traffic and it is at the junction of the major influent of the River Tiber, the river Aniene, which in turn receives the wastewaters of the industrial area around Rome. Point 4 is located at the basin closure of the River Tiber, receiving the total urban discharges; moreover, this stretch of the river is navigable, so that small quantities of oil spill could contribute as well to the variations in PAH concentrations.

### 3.5 Polar organic contaminants (pesticides, biocides, insect repellents, corrosion inhibitors, pharmaceuticals, and PFAS)

**Fig. 6:**
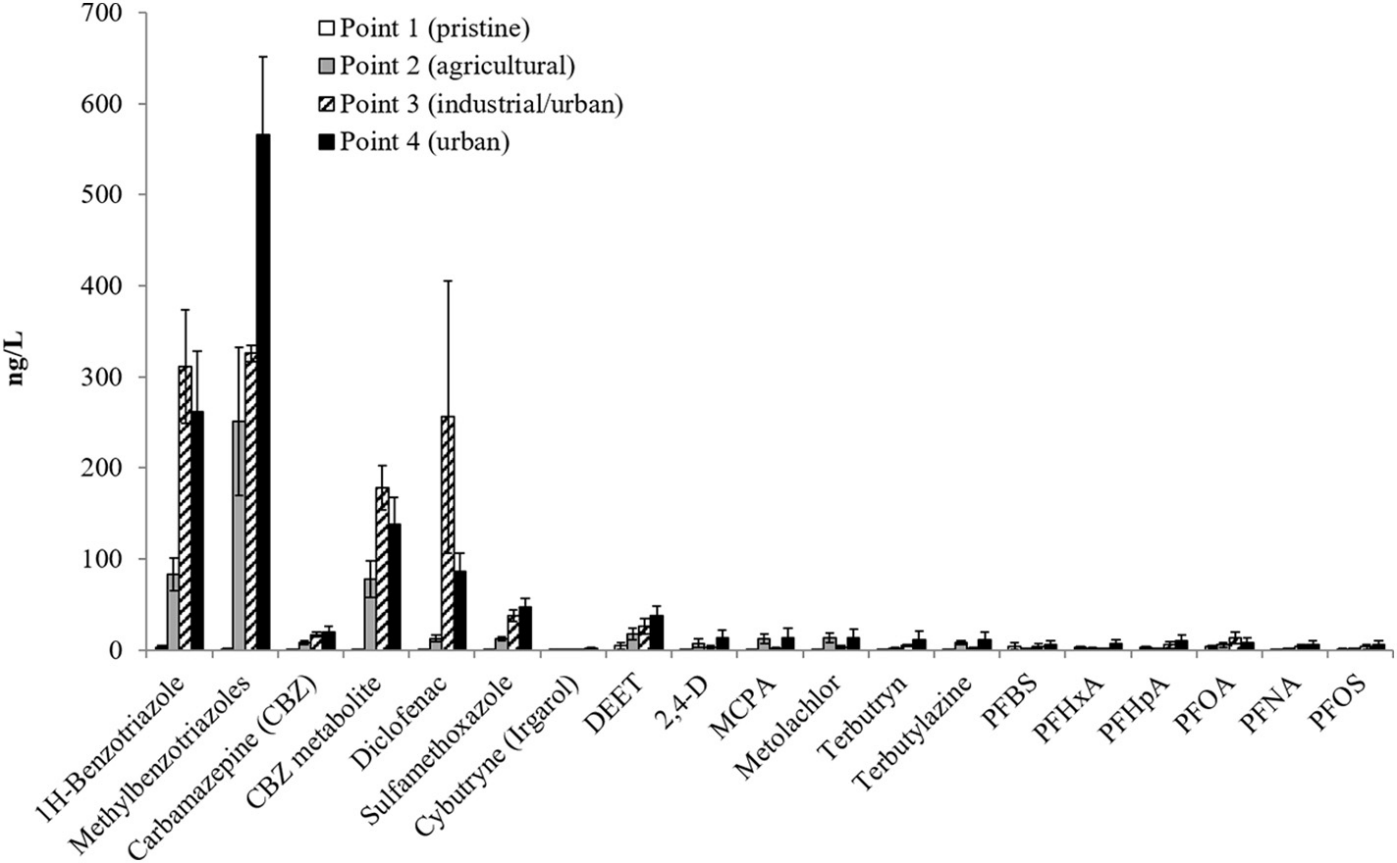
Polar organic compounds. Average concentrations (ng/L) of the five samplings in the four points (1, 2, 3 and 4) of the River Tiber. The vertical bars represent the standard errors.

The analytical results of the polar organic compounds are reported in Table SI6. Fig. 6 shows the average concentrations of the four sampling campaigns. The highest concentrations were found for the benzotriazoles (up to 852 ng/L for methybenzotriazole at point 4), 10,11-dihydro-10,11-dihydroxy-carbamazepine (a persistent hydroxy-metabolite of the mood-stabilizing drug carbamazepine; up to 236 ng/L at point 4 and 225 ng/L in the River Aniene), and the non-steroidal anti-inflammatory drug diclofenac (max. 849 ng/L; point 3 River Aniene). A high temporal variation was observed for diclofenac, benzotriazoles, carbamazepine metabolite, and sulfamethoxazole at points 3 and 4 in the last sampling of October 2015 due to heavy rain falls before sampling, causing high water flows and likely overflows of the WWTPs. The diclofenac concentration increased from levels between 49 and 159 ng/L in the previous samplings to 849 ng/L (at point 3). Launay et al. (2016) showed that the emissions from combined sewer overflows (CSOs) contribute to the discharge of a wide range of organic micro-pollutants from wastewater systems to urban receiving waters. Low concentrations of pesticides were observed in the agricultural area (point 2). Maximum concentrations of around 30 ng/L were detected for 2,4-D, MCPA and metolachlor. Slightly higher (average) pesticide levels were observed at the river mouth (Fiumicino; point 4): 12.7 ng/L for 2,4-D, 37.7 ng/L for DEET, 13.2 ng/L for metolachlor, and 11.5 ng/L for terbutryn and 11.3 ng/L for terbutylazine.

Benzotriazoles, pharmaceuticals, and perfluoroalkyl substances (PFAS) were particularly found at the sampling points 3 (River Aniene, downstream from Rome East WWTP) and 4 (River Tiber close to the river mouth, downstream from the urban WWTP of Southern Rome). In spring 2014, several PFAS were detected in a concentration range of 20 ng/L at point 4 (Table SI6). Furthermore at the same point and period higher concentrations were detected as well for 2,4-D (47 ng/L), carbamazepine (46 ng/L), cybutryne (4.5 ng/L), MCPA (55 ng/L), metolachlor (53 ng/L), terbutryn (46 ng/L), and terbutylazine (44 ng/L), resulting overall the most polluted site and time. Along with this result high concentrations for pyrene, benzo(a)pyrene, iron, manganese, and aluminum in the same sample were measured.

The EQS were only exceeded for perfluorooctansulfonate (PFOS) (EQS: 0.65 ng/L), diclofenac (EQS: 100 ng/L; average 256 ng/L at point 3 and 86 ng/L at point 4), and cybutryne (EQS: 2.5 ng/L; at point 4 2.9 ng/L in October 2013). Some samples were contaminated (“con.”) during sample preparation in the laboratory for PFAS (Table SI6). The EQS of PFOS was exceeded at points 2, 3, and 4 in October 2013 and March 2014. In October 2014, April 2015, and October 2015 its concentration was ≤1.1 ng/L (the LOQ (1.1 ng/L) was due to a blank problematic of the analytical procedure above the EQS of 0.65 ng/L). The highest PFOS levels were detected in March 2014 at point 3 (14 ng/L) and point 4 (24 ng/L).

### 3.6 Microbial abundance, *Bacteria*, *Archaea*, coliforms, *E. coli* and *Enterococcus* spp.

The total microbial abundance and the percentage of the main microbial domains (*Bacteria* and *Archaea*) detected at the four sampling points, reflected the natural river characteristics. Indeed, a general tendency of microbial abundance increase from the source (2.39 × 10^5^ cells/mL) to the mouth (1.57 × 10^6^ cells/mL) of the river was confirmed (Table SI7). Along the river course, *Bacteria* and *Archaea* domain accounted for 12–73% and 0–2% of total cells, respectively, with values comparable to those found in other lotic waters (Araya et al., 2003; Bouvier and Del Giorgio, 2003; Grenni et al., 2014).

Microbial abundance, cell viability and number of cells of *Bacteria* domain differed significantly between sampling points (P < 0.005), but this was neither influenced by seasonality nor by sampling campaigns (P > 0.05). On the contrary, the *Archaea* domain showed significantly higher values in autumn compared to spring (P < 0.01), but did not significantly change among points. An overall low microbial abundance was observed in the spring 2015 campaign, throughout the entire river course.

Additional parameters as indicators of anthropogenic pressure were measured, i.e. the total Coliforms, *E. coli* and *Enterococcus* spp. (Table SI7). In October 2013, the former selected pristine point Pieve di Santo Stefano showed an unexpected fecal coliform occurrence (total coliforms 1568 MPN/100 mL), low cell viability (7.1% live cells), and relatively high ion concentrations, together with elevated PAHs levels (∑PAHs 38.7 ng/L) (Tables SI2, SI4 and SI7). An additional sampling, performed in February 2014, confirmed that the fecal contamination in Pieve di Santo Stefano presumably due to grazing animals and a farm presence in the surrounding area was not occasional (data not shown), and a new pristine point was selected, Monte Fumaiolo, based on absence of fecal indicator and higher cell viability values.

### 3.7 Bacterioplankton phylogenetic composition

**Fig. 7:**
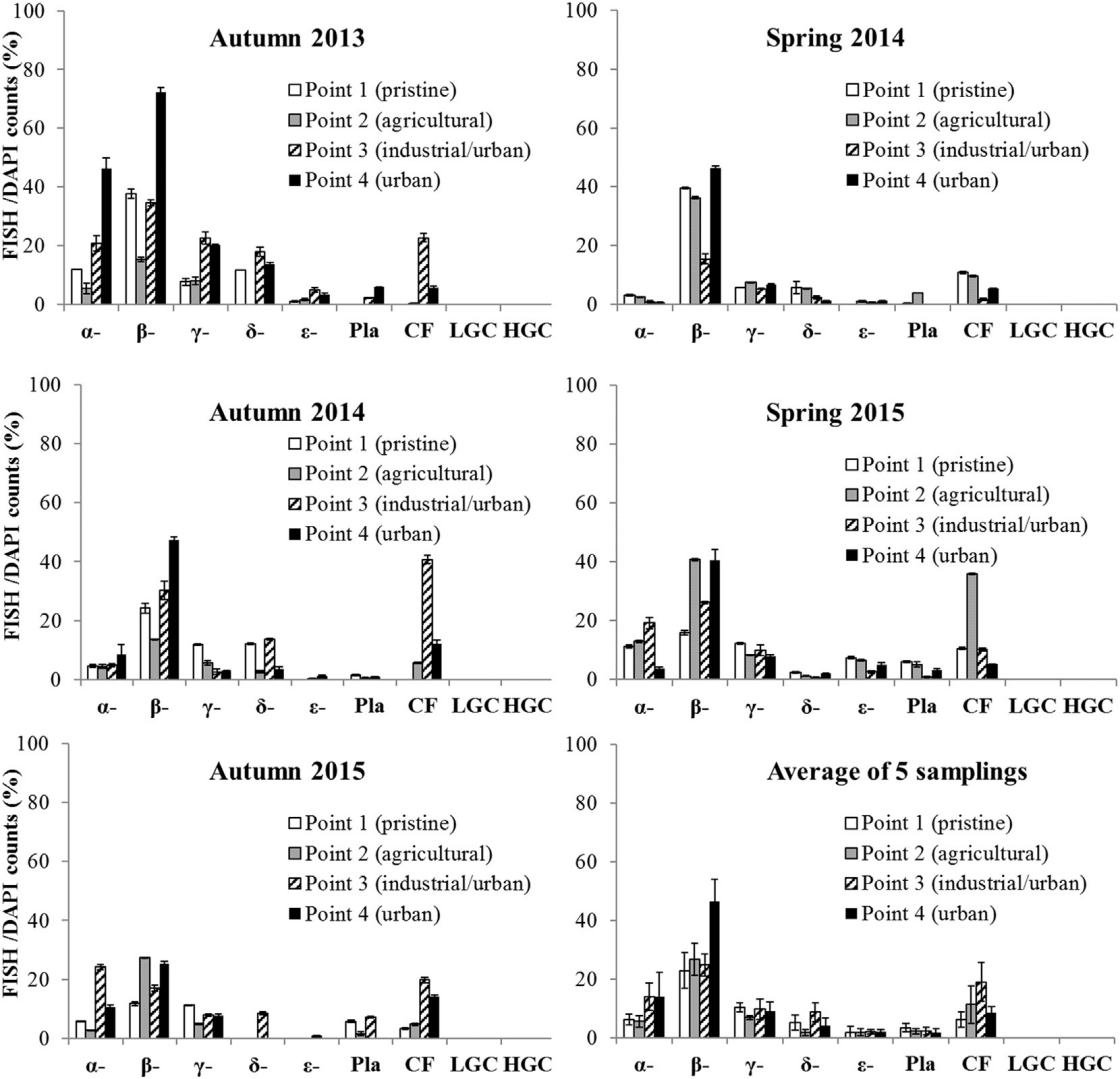
Bacterial phylogenetic composition analyzed by FISH. α-: α-*Proteobacteria*; β-; β-*Proteobacteria*; γ-: γ-*Proteobacteria*; δ-: δ-*Proteobacteria*; ε-: ε-*Proteobacteria*; Pla: *Planctomycetes*; CF: *Bacteroidetes*; LGC: *Firmicutes*; HGC: *Actinobacteria*. Cells binding to each probe were estimated as % of the total DAPI positive cells. The vertical bars represent the standard errors.

The phylogenetic characterization of the bacterial community within the *Bacteria* domain showed that α-, β-, γ-, δ-, ε-*Proteobacteria* represented the majority of bacterial populations at all four sampled points (Fig. 7). β-*Proteobacteria*, CF (*Bacteroidetes formerly Cytophaga-Flavobacterium-Bacteroides*) and α-*Proteobacteria*, in decreasing order were primarily responsible for the observed differences between points, as indicated by SIMPER analysis. β-*Proteobacteria* were overall the most abundant class and differed significantly between the points (P < 0.005) and not between seasons (P > 0.05). They were constantly high in the urban area (point 4), with a mean value of 19% higher than in all other points.

The second most abundant bacterial group was the *Bacteroidetes* phylum, formerly known as the Cytophaga-Flavobacterium-Bacteroides (CF). An association of *Bacteroidetes* with the industrial point 3 in the autumn samplings was observed (Fig. 7, Fig. 9b). Indeed, in the autumn 2014 campaign, 41% of *Bacteria* at point 3 were classified as *Bacteroidetes*, with the highest value observed throughout all campaigns.

The α-*Proteobacteria* class was the third most represented group. This class differed significantly by seasonality (P < 0.05), showing lower values in spring, and higher ones in the industrial and urban points (points 3 and 4) as compared to the other points (with a mean value of 14% of *Bacteria* in both points).

γ-*Proteobacteria*, ranging from 7 to 10% of *Bacteria*, differed significantly by points (P < 0.05), but not by seasonality. The bacterial taxa found in minor percentages (δ - *Proteobacteria*, ε - *Proteobacteria* and *Planctomycetes*), did not differ significantly by none of the factors analyzed.

The gram-positive bacteria *Firmicutes* (LGC) and *Actinobacteria* (HGC) were detected in negligible concentrations (below 5 cells in 30 fields under the epifluorescence microscope).

**Fig. 8:**
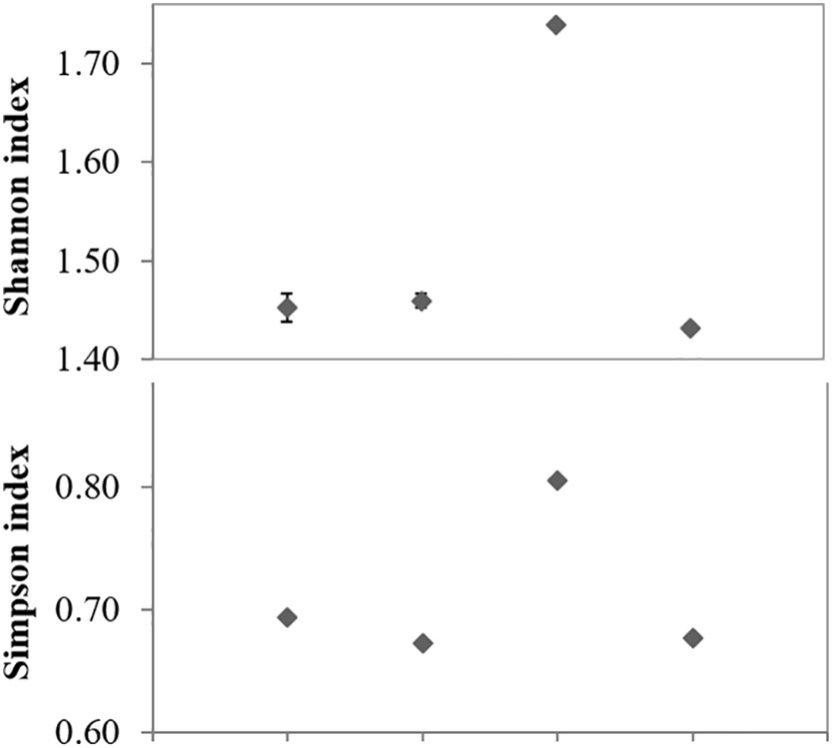
Shannon and Simpson Diversity indices inferred from the FISH analysis of the bacterial community in the four sites of the River Tiber, Point 1 (pristine), Point 2 (agricultural), Point 3 (industrial/urban), Point 4 (urban).

The results of the calculation of the Shannon and Simpson diversity indices using the data obtained by the FISH analysis shows significantly higher values in point 3 than in other points (Fig. 8). The Shannon index takes into account the number of individuals (bacterial cells) and of taxa, whereas Simpson expresses how evenly the individuals are distributed within the different species (evenness).

### 3.8 Principal Component Analysis (PCA)

**Fig. 9:**
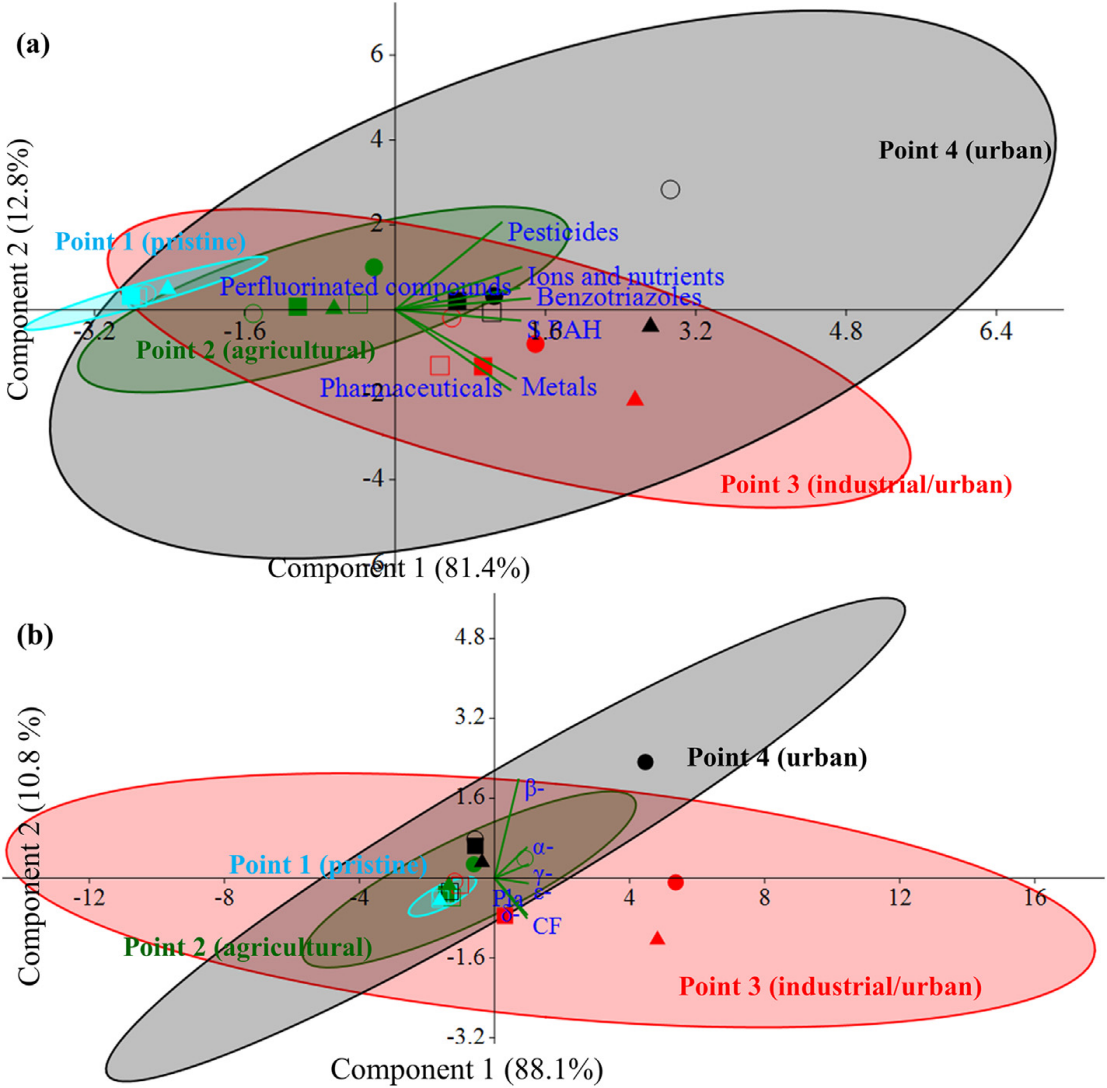
Principal component analysis (PCA) between-sites inferred from chemical parameters (a) and from microbial community structure (b). Different symbols correspond to the five samplings: filled symbols indicate autumn samplings and empty symbols spring samplings. Ellipses estimate the regions where 95% of points are expected to fall. α-: α-*Proteobacteria*; β-: β-*Proteobacteria*; γ-: γ*-Proteobacteria*; δ-: δ-*Proteobacteria*; ε-: ε-*Proteobacteria*; Pla: *Planctomycetes*; CF: *Bacteroidetes*.

PCA of grouped chemical parameters (ions and nutrients, metals, ΣPAH, benzotriazoles, pharmaceuticals, pesticides, perfluorinated compounds) (Table 1), measured in the five sampling campaigns, was inferred between sampling points (Fig. 9a). Most of the variance was explained by component 1 (81.4%) and in minor percentage by component 2 (12.8%), indicating significance of the test (Fig. 9a). The four points divided across the PCA quadrants, with pristine point 1 in the left side of the biplot, indicating a negative correlation with component 1, representing most of the analyzed chemicals. All samplings in pristine point were close to each other, indicating high similarity of chemical data among campaigns. The agricultural point 2 fell in an intermediate position between pristine and the other two points, suggesting a scarce association with the analyzed compounds. On the contrary, the chemical parameters were related with the industrial/urban and urban points. In particular, the urban point was associated with pesticides, ions and nutrients and benzotriazoles (Fig. 9a). The industrial/urban point 3 was mostly related to pharmaceuticals and metals (Fig. 9a).

Variance in the PCA analysis inferred from microbial community composition between sampling points was also explained mainly by the first two components (respectively 88.1% and 10.8%) (Fig. 9b). Industrial/urban point 3 was linked to the CF, whilst the urban point 4 to the β-*Proteobacteria* (Fig. 9b), in accordance with the highest concentration of these classes, observed in these points.

## 4 Discussion

The overall chemical results indicated a correspondence between different groups of contaminants found and the main contamination sources of the selected sampling areas. The absence of chemical and biological contamination at the river source (Monte Fumaiolo) confirmed the choice of substituting the upstream point (Pieve di Santo Stefano) due to the detection of fecal contamination indicators.

A general low concentration of pesticides, corrosion inhibitors and PAHs was found in the agricultural area indicating a scarce pollution of the river at this point due to agricultural practices. Nevertheless, the relatively high levels of metals such as Ba, Al and Fe indicated a source of industrial contamination (Sawai et al., 2016). In fact, because there is a foundry close to this point, the occurrence of these metals can be ascribed to releases from it.

The high levels of Sr, As and U found at industrial point 3 can be explained by the authorized discharge into the river of groundwater and thermal water (used for the extraction of travertine quarries) containing these metals naturally. They are accordingly of geogenic origin and their values are comparable with those found in other studies in the same area or in thermal water (Minissale et al., 2002; Carucci et al., 2012; Cinti et al., 2015). In particular, As (which was the only one above the Italian legal limit) can be found naturally in concentrations up to 62 μg/L in the water bodies of central Italy (Cinti et al., 2015).

Relatively low concentrations of PAHs were found in our study (max. 65 ng/L at point 4) compared to those previously reported by other authors in the same river (Minissi et al., 1998; Patrolecco et al., 2010). Montuori et al. (2016) reported higher PAHs levels in River Tiber, with a total PAH concentration in the dissolved water phase in Fiumicino of 607 ng/L (70 ng/L for the 5-ring PAHs benzo(b)fluoranthene, benzo(k)fluoranthene, benzo(a)pyrene, and dibenzo(a,h)-anthracene, and 35 ng/L for the 6-ring PAHs benzo(g,h,i)perylene, indeno(1,2,3-cd)pyrene).

High concentrations of pharmaceuticals, such as the hydroxy-metabolite of the mood-stabilizing drug carbamazepine and the non-steroidal anti-inflammatory drug diclofenac were present at sampling points 3 and 4, which are close to the WWTPs. Point 4 was the most polluted one, particularly in spring 2014, when the highest concentrations were observed for the polar compounds such as PFAS, pharmaceuticals and pesticides, as well as for some PAHs.

The microbiological parameters, such as the total microbial abundance and the percentages of the main microbial domains (*Bacteria* and *Archaea*), detected at the four sampling points, reflected both the natural river characteristics (e.g. a general tendency of bacterial abundances to increase from the source to the mouth of the river) and presence of contaminants. The phylogenetic analysis of the bacterial community revealed differences among the sampling points, which could reflect their adaptation to the different anthropogenic pressures. β- and α-*Proteobacteria* and *Bacteroidetes* were the most abundant bacterial groups in the river, as found by other authors (Araya et al., 2003; Kirchman, 2002; Zwart et al., 2002).

At all points, the dominant group was the β-*Proteobacteria*. Indeed, this class often represents a high proportion of planktonic *Bacteria* in lakes, rivers, reservoirs, lotic biofilms and WWTPs (Becerra-Castro et al., 2016; Parveen et al., 2011; Pernthaler, 2013; Xu et al., 2018; Zwart et al., 2002).

β-*Proteobacteria* were the highest class in urban point 4 (Fig. 7 and Table SI8). An association of this point with ions and nutrients, benzotriazoles and pesticides was observed (Fig. 9a). The high load of nutrients discharged from the WWTPs into the final points of the river and the contaminant occurrence may be a favorable habitat for β-*Proteobacteria* populations. The dominance of β-*Proteobacteria* in river water has been attributed to their ability to degrade organic pollutants and to oxidize ammonia (Araya et al., 2003). Changes in numbers and species of β-*Proteobacteria* ammonia oxidizers have been postulated as in situ indicators of the biological impact of pollutants (Kowalchuk and Stephen, 2001). Moreover, a relationship between the abundance of β-*Proteobacteria* and the degree of pollution has been previously hypothesized (Brümmer et al., 2003). Indeed, an association between the β-*Proteobacteria* and benzotriazole biodegradation has been reported in activated sludge (Herzog et al., 2013). Furthermore, several species of β-*Proteobacteria* able to degrade 2,4-D, MCPA, metolachlor, terbuthylazine, other pesticides (Barra Caracciolo et al., 2010; Liu et al., 2011) and also PAHs (Ghosal et al., 2016; Singleton et al., 2009) have been found. These preliminary results can be used to further elucidate which specific bacteria, within the β-*Proteobacteria* class, may be used as bioindicators of multiple stress in river water.

Although FISH method makes it possible to evaluate the bacterial diversity at high taxonomic ranks, it gave us, as a first approach, an overall information on the diversity of each sampling site. The markedly higher bacterial diversity observed at point 3 (Fig. 8) is in accordance with a higher diversity of bacterial communities observed in river samples downstream from a WWTP in other studies (Wakelin et al., 2008; Marti et al., 2013). The presence at point 3 of different anthropogenic pressures (i.e. WWTP and the industrial ones) at an intermediate level (the legal limits of pollutants were indeed exceeded only in few cases and the overall contamination was lower than the point 4), may have exerted a positive pressure on bacterial biodiversity. Indeed, in addition to the highest average values of bacterial viability, the highest number of bacteria hybridizing with the rRNA probes was also found here, indicating a higher physiological activity of bacteria at this point. An adaption of the local bacterial populations to the chemical status at this point, in terms of resistance and/or capacity to degrade pollutants, may be hypothesized. At this point α-*Proteobacteria* were higher than at the other points, and displayed a positive correlation with pharmaceuticals (r = 0.75; P = 0.0006) (Table SI9). Interestingly, this correlation was significant also in point 4 (r = 0.75; P = 0.0005). Antibiotic resistance, Strontium tolerant, natural recalcitrant and anthropogenic compound degradation have been reported among α-*Proteobacteria* (Falcone-Dias et al., 2012; Ozer et al., 2013; Stolz, 2009; Thelusmond et al., 2016), indicating a certain metabolic diversity within these microorganisms. The *α-Proteobacteria* include several cosmopolitan freshwater species able to tolerate nutritionally rich environments, as is the case of the last stretch of the River Tiber.

At points 3 and 4, pharmaceuticals were also correlated with the *Bacteroidetes* phylum (r = 0.78; P = 0.0002 in both points). This phylum constitutes a significant proportion of the microbial communities in aquatic environments and *Bacteroidetes* members are specialized in the degradation of complex macromolecules. Examples include their high antibiotic multi-resistance (Falcone-Dias et al., 2012; Xiong et al., 2015), their increase in riverine biofilm communities in response to ibuprofen treatment (Lawrence et al., 2005), their dominance in activated sludge (Muter et al., 2017) and in hospital wastewater (Prasertkulsak et al., 2016). An increase of *Bacteroidetes* in biofilms in a polluted Spanish river, together with a positive correlation with the antibiotic tetracycline has been observed (Proia et al., 2013). Moreover, a high percentage of *Bacteroidetes* was isolated from plants exposed to carbamazepine and able to uptake this pharmaceutical from liquid medium (Sauvetre and Schroder, 2015). The presence of drug-resistant or -degrading species belonging to this phylum in the final stretch of the River Tiber cannot, therefore, be excluded.

At these two sites, significant correlation was also found between metals and the *Bacteroidetes* (r = 0.73; P = 0.0009 and r = 0.73; P = 0.0008 respectively in point 3 and 4). Among metals, As-resistant bacteria belonging to this phylum have been found in freshwaters at the same area (Davolos and Pietrangeli, 2013). Furthermore, microbial communities associated with U-contaminated environments have been found to also include *Bacteroidetes* among the most abundant bacterial taxa (Rastogi et al., 2010).

In a dynamic system such as a river basin, establishing a causal relationship between chemical contamination and microbial community responses is a major challenge that needs specific laboratory assays to be evaluated (Tlili et al., 2016). Nevertheless, since exposure of a natural community to a pollutant favours the disappearance of sensitive populations and the dominance of tolerant ones, the positive correlations of the α-Proteobacteria and Bacteroidetes with mixtures of pollutants may indicate the presence of tolerant species among these populations.

The negligible detection of the gram positive bacteria *Firmicutes* and *Actinobacteria*, even though they are reported to inhabit freshwaters, may be due to the fact that the robust cell wall or the formation of endospores might have affected the probes' entrance inside the cells. Moreover, we cannot exclude that a low activity of these phyla, and consequently a low rRNA content, may have contributed to the low fluorescence signals under the microscope. Indeed, full coverage of bacterial cells hybridized with rRNA-targeted probes is rarely obtained because it is directly related to the metabolic state of the cells and the low ribosome content of slowly growing cells may affect the detection efficiency of probes.

In this study the FISH analysis was used at first glance to investigate the correlation of different anthropogenic pressure with the microbial community, the metagenomics analysis is the step forward since it helps to provide a more comprehensive and deeper knowledge of the microbial composition and its functioning linked to the environmental stressors. Currently these experiments are under consideration.

## 5 Conclusions

Microbial communities respond and adapt quickly to environmental conditions. They reflect the water profile providing then a snapshot of water quality. In this study a correlation between some microbial taxa (e.g. β-, α-*Proteobacteria*, *Bacteroidetes*) and the complexity of chemical pollutants was observed, suggesting that microorganisms can be useful bio-indicators of water quality. Consequently, they should be considered in the regulatory process i.e. the Water Framework Directive bridging the current ecological status with chemical status. The use of this approach is particularly effective because it takes into consideration the overall effect of contaminant mixtures independently from the concentration of each single contaminant and may also help in identifying possible synergistic effects. Indeed only a holistic approach can contribute to addressing the water quality and it should be designed taking into account the microbes and their functioning.

## Appendix A Supplementary data

Supplementary material Image 1
