# Supplementary material for: Chemical mixtures and fluorescence in situ hybridization analysis of natural microbial community in the Tiber river
Source: Sci Total Environ. 2019 Jul 10;673:7–19. doi: 10.1016/j.scitotenv.2019.04.011 (PMC6509555; doi:10.1016/j.scitotenv.2019.04.011)
Supplement: Supplementary file 1 — Supplementary material [file mmc1.doc]

**CHEMICAL MIXTURES AND FLUORESCENCE IN SITU HYBRIDIZATION ANALYSIS OF NATURAL MICROBIAL COMMUNITY IN THE TIBER RIVER**

Maria Ludovica Saccà^1,5$^, Valentina Elisabetta Viviana Ferrero^2$^, Robert Loos^3^, Martina Di Lenola^1^, Simona Tavazzi^3^, Paola Grenni^1^, Nicoletta Ademollo^1^, Luisa Patrolecco^1^, Jim Huggett^2,4^, Anna Barra Caracciolo^1^ and Teresa Lettieri^3*^

^1^National Research Council, Water Research Institute, Via Salaria km 29,300, 00015 9 Monterotondo (Rome) – Italy

^2^Molecular and Cell Biology team, LGC, Queens Road, Teddington, Middlesex, TW11 0LY, United Kingdom

^3^European Commission, Joint Research Centre (JRC), Ispra, Italy

^4^School of Biosciences & Medicine, Faculty of Health & Medical Science, University of Surrey, Guildford, Surrey GU2 7XH, United Kingdom

^5^Present address: Council for Agricultural Research and Economics, Research Center for Agriculture and Environment (CREA - AA), Via di Corticella 133, Bologna - Italy.

^$^ Equal contribution

* Author to whom all correspondence should be addressed: E-mail: [teresa.lettieri@ec.europa.eu](mailto:teresa.lettieri@ec.europa.eu)

**Supplementary material**

### Sampling points

Pictures of the four sampling points (Tiber and Aniene Rivers).

**1.1.** **Monte Fumaiolo**

Pristine sampling point 1 (43°47'11''N, 12°04'50''E) at the source of the Tiber river in Emilia-Romagna region.


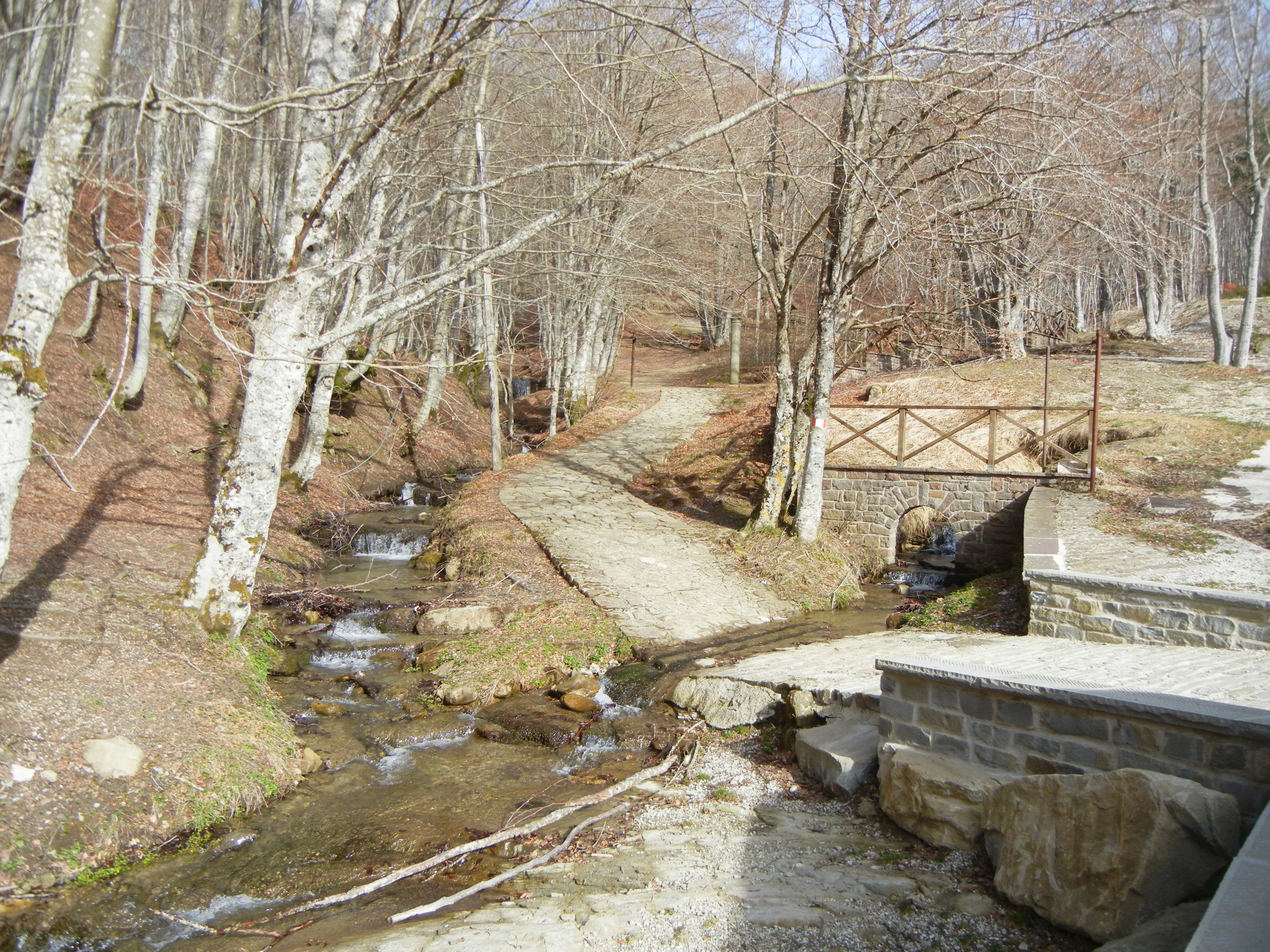


### Attigliano

Agricultural sampling point 2, in the Province of Terni, Umbria region (42°30'30''N, 12°16'59''E).


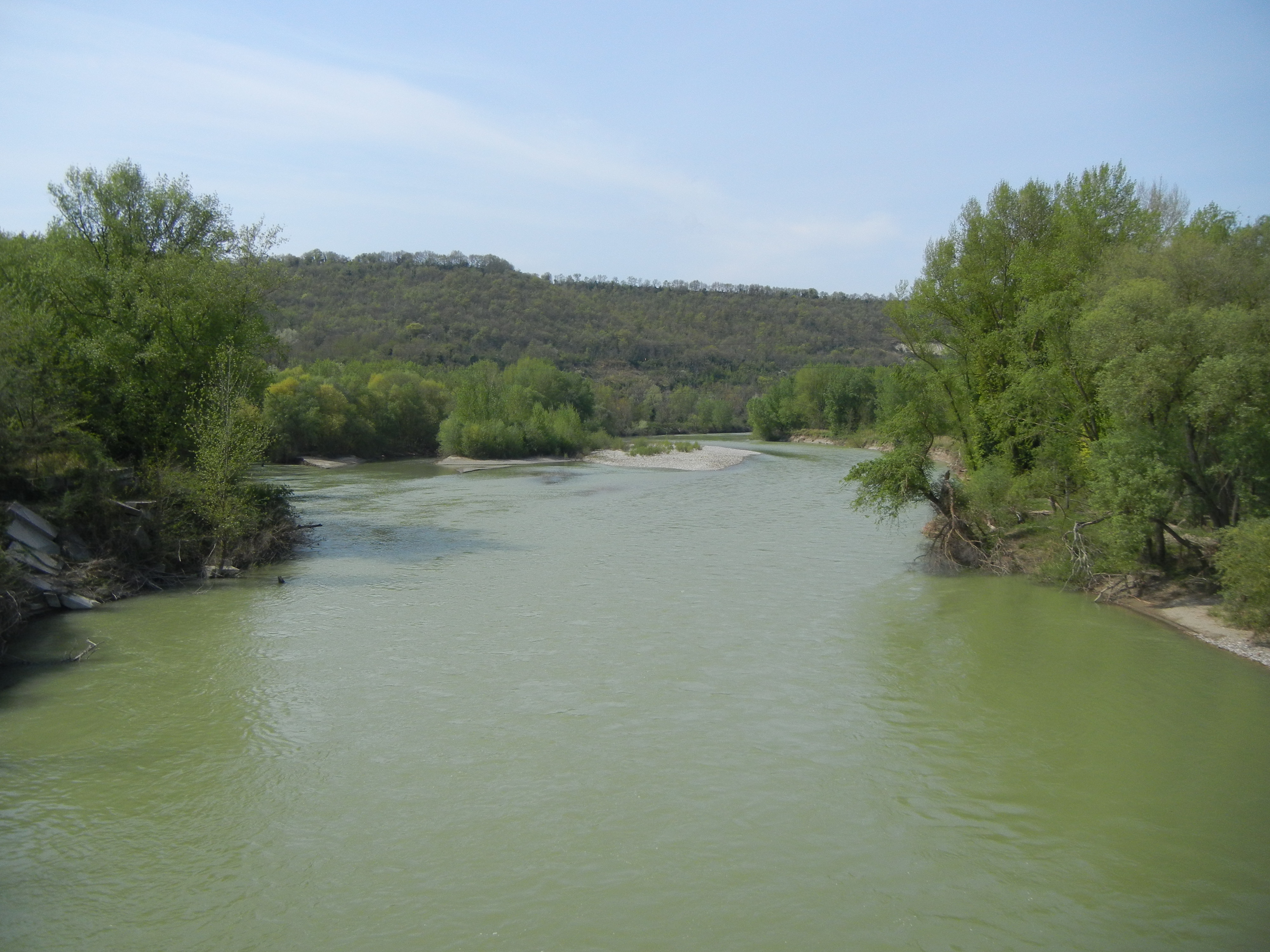


### 1.3. River Aniene

Industrial and urban sampling point 3, where River Aniene flows into the River Tiber in Rome (41°56'22''N, 12°30'26''E), downstream from Rome East wastewater treatment plant (WWTP).


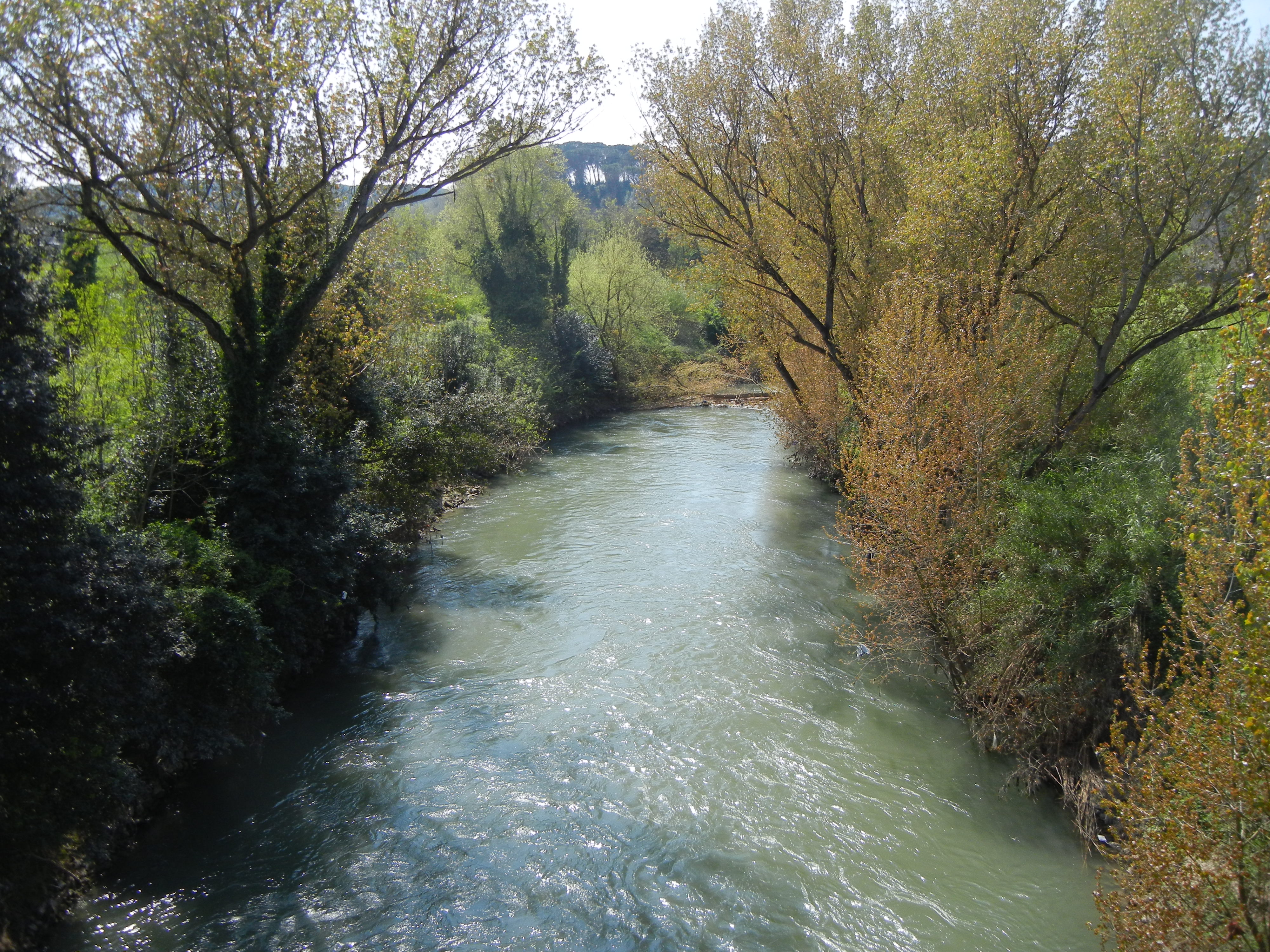


### Fiumicino

Urban sampling point 4, downstream from the Southern WWTP of Rome and close to the river mouth (41°48'15''N, 12°14'50''E).


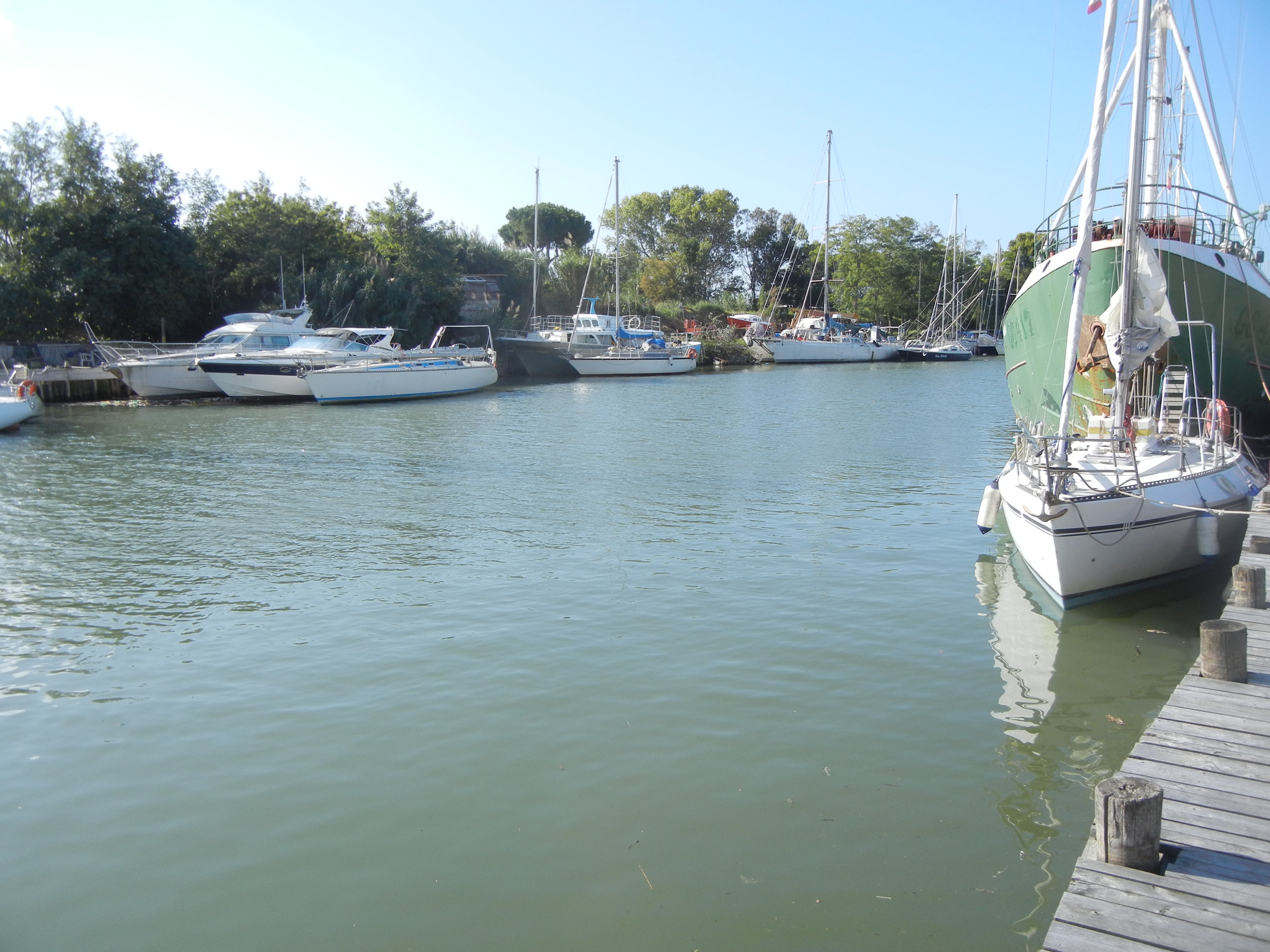


### 2. Selection of the polar organic target compounds

Cybutryne (a biocide, also known as irgarol, which is mainly used as an antifouling agent in paints for boats and vessels and which is applied at marine as well as at inland freshwater sites), terbutryn (an herbicide and biocide or algicide used in paints) and PFOS are new priority substances under the WFD (Directive 2013/39/EU; EU, 2013). Diclofenac, a non-steroidal anti-inflammatory drug used to treat pain, was proposed as a new priority substance in 2012 (with a proposed EQS of 0.1 µg/L; note that the Swiss Ecotox Centre proposes a lower Predicted No Effect Concentration-PNEC of 0.05 µg/L), and is monitored under the newly introduced WFD ‘watch list’ mechanism (EU, 2013; Tiedeken et al., 2017). Carbamazepine, a mood-stabilising drug used for the treatment of epilepsy, bipolar depression, excited psychosis, mania, and sleeping disorders, is one of the pharmaceuticals most often analysed in the environment, and 10,11-dihydro-10,11-dihydroxy-carbamazepine (CBZ-diOH) an important degradation product of carbamazepine for which only few monitoring data are available (De Laurentiis et al., 2012; Fenet et al., 2012; Hummel et al., 2006; Miao and Metcalfe, 2003). In addition, the sulfonamide antibiotic sulfamethoxazole, used to treat urinary tract infections, sinusitis and toxoplasmosis, is often detected in the aqueous environment (Johnson et al., 2015; Straub, 2016; Patrolecco et al., 2018) and, along with the herbicides 2,4-D, MCPA and metolachlor (because frequently detected; Schreiner et al., 2016) and the insect repellent DEET (Costanzo et al. 2007), was included in the study. Benzotriazoles (1H-benzotriazole and 5-methyl-1H-benzotriazole) were chosen because they are produced in high volume (being used as corrosion inhibitors) and detected in industrial and urban areas (Weiss et al., 2006) but were analysed in only selected samples in JDS2 (Loos et al., 2010). Note that 4- and 5-methyl-1H-benzotriazole cannot be separated chromatographically and, therefore, only the total concentration of methylbenzotriazoles is given in the results. In addition, several perfluoroalkyl substances were analysed because they were found to be important pollutants in Italy (Valsecchi et al., 2015).

### Tables

**Table SI1: Physico-chemical parameters.**

Values in red highlight higher or lower than average temporal concentrations (within a sampling point). Values in **orange** highlight higher or lower than average spatial concentrations (between sampling points; explained in the text). Average of sampling campaigns ± standard error is reported in the last column. n.a.: not applicable.

| **Point 1 - Pristine** | ***Oct-13*** | **Mar-14** | **Oct-14** | **Apr-15** | **Oct-15** | **Average^1^** |
| --- | --- | --- | --- | --- | --- | --- |
| **Temperature (°C)** | *13.3* | 5.0 | 10.3 | 7.0 | 9.9 | 8.1±1.3 |
| **pH** | *7.9* | 7.9 | 7.9 | 7.8 | 7.0 | 7.7±0.2 |
| **Redox Potential (mV)** | *236* | 252 | 275 | 173 | 590 | 323±91.8 |
| **Conductivity (µS/cm)** | *560* | 272 | n.a. | 285 | 216 | 258±18.4 |
| **Dissolved oxygen saturation (%)** | *95.0* | 101.7 | 97.3 | 93.1 | 87.5 | 94.9±3.0 |
| **Dissolved oxygen (mg/l)** | *10.1* | 11.1 | 9.4 | 9.9 | 8.5 | 9.7±0.5 |

^1^: Sample from Oct-13 excluded (see text).

| **Point 2 - Agricultural** | **Oct-13** | **Mar-14** | **Oct-14** | **Apr-15** | **Oct-15** | **Average** |
| --- | --- | --- | --- | --- | --- | --- |
| **Temperature (°C)** | 16.5 | 11.4 | 20.0 | 13.3 | 20.4 | 16.3±1.8 |
| **pH** | 7.9 | 7.9 | 7.8 | 7.9 | 7.2 | 7.7±0.1 |
| **Redox Potential (mV)** | 238 | 245 | 257 | 249 | 548 | 307±60.2 |
| **Conductivity (µS/cm)** | 567 | 125 | 644 | 631 | 605 | 514±98.2 |
| **Dissolved oxygen saturation (%)** | 83.8 | 104.2 | 100.2 | 103.0 | 92.2 | 96.7±3.8 |
| **Dissolved oxygen (mg/l)** | 8.2 | 11.2 | 9.0 | 10.8 | 8.2 | 9.5±0.6 |

| **Point 3 – Industrial/Urban** | **Oct-13** | **Mar-14** | **Oct-14** | **Apr-15** | **Oct-15** | **Average** |
| --- | --- | --- | --- | --- | --- | --- |
| **Temperature (°C)** | 16.4 | 11.8 | 17.2 | 14.8 | 18.4 | 15.7±1.1 |
| **pH** | 7.2 | 7.3 | 7.2 | 7.0 | 7.1 | 7.2±0.1 |
| **Redox Potential (mV)** | 196 | 258 | 177 | 149 | 340 | 224±33.9 |
| **Conductivity (µS/cm)** | 1495 | 326 | n.a. | 875 | 1273 | **992**±229.2 |
| **Dissolved oxygen saturation (%)** | 53.0 | 76.7 | 65.6 | 71.8 | 49.4 | **63.3**±5.3 |
| **Dissolved oxygen (mg/L)** | 5.2 | 8.2 | 6.2 | 7.3 | 4.6 | **6.3**±0.7 |

| **Point 4 - Urban** | **Oct-13** | **Mar-14** | **Oct-14** | **Apr-15** | **Oct-15** | **Average** |
| --- | --- | --- | --- | --- | --- | --- |
| **Temperature (°C)** | 16.8 | 12.7 | 18.0 | 14.5 | 18.9 | 16.2±1.1 |
| **pH** | 7.5 | 7.3 | 7.4 | 7.3 | 6.8 | 7.3±0.1 |
| **Redox Potential (mV)** | 225 | 238 | 229 | 96 | 440 | 246±55.3 |
| **Conductivity (µS/cm)** | 1320 | 1021 | 2522 | 994 | 5261 | **2223**±809.0 |
| **Dissolved oxygen saturation (%)** | 68.8 | 90.9 | 60.5 | 78.8 | 47.0 | **69.2**±7.5 |
| **Dissolved oxygen (mg/L)** | 6.3 | 9.5 | 5.6 | 8.1 | 4.3 | **6.8**±0.9 |

**Table SI2: Results for Metals.** Concentrations in (μg/L).

AA-EQS = annual average EQS; MAC-EQS = maximum allowable concentration EQS. Values in red highlight higher or lower than average temporal concentrations (within a sampling point; explained in the text). Values in **orange** highlight higher or lower than average spatial concentrations (between sampling points). Values in **dark red bold**: EQS exceedance. Values < LOQ were considered half of the LOQ for average calculation. Average of sampling campaigns ± standard error is reported in the last column. n.a.: not applicable, the chemical is not reported in any list with an EQS value (see paragraph 2.6 of the main text).

| **Point 1 – Pristine** | **AA-**  **EQS** | **MAC-EQS** | ***Oct-13*** | **Mar-14** | **Oct-14** | **Apr-15** | **Oct-15** | **Average^1^** |
| --- | --- | --- | --- | --- | --- | --- | --- | --- |
| **Ba** | 73 | n.a. | ***94*** | 19 | 22 | 23 | 21 | 21±1.0 |
| **Sb** | 5.6 | n.a. | *0.1* | <LOQ | <LOQ | <LOQ | <LOQ | 0.02±0.0 |
| **As** | 10 | n.a. | *0.2* | <LOQ | <LOQ | <LOQ | <LOQ | 0.02±0.0 |
| **Cd** | 0.2 | 0.45 | <LOQ | <LOQ | <LOQ | <LOQ | <LOQ | 0.02±0.0 |
| **Cr** | 2.5 | 7 | *0.3* | 0.2 | 0.5 | 0.8 | 1.1 | 0.7±0.2 |
| **Cu** | 7.8 | n.a. | *1.4* | <LOQ | 0.4 | 0.2 | 0.3 | 0.2±0.1 |
| **Pb** | 1.2 | 14 | *0.2* | <LOQ | <LOQ | <LOQ | <LOQ | 0.02±0.0 |
| **Hg** | 0.05 | 0.07 | <LOQ | <LOQ | <LOQ | <LOQ | <LOQ | 0.02±0.0 |
| **Ni** | 4 | 34 | *1.8* | <LOQ | <LOQ | <LOQ | <LOQ | 0.02±0.0 |
| **Se** | 1 | 10 | ***1.2*** | <LOQ | <LOQ | <LOQ | <LOQ | 0.02±0.0 |
| **V** | 3.5 | 50 | *0.3* | <LOQ | <LOQ | <LOQ | 0.1 | 0.04±0.02 |
| **Fe** | 200 | n.a. | *9.8* | 8.5 | 5.7 | 6.1 | 7.8 | 7.0±0.7 |
| **Zn** | 20.6 | n.a. | *9.8* | 0.2 | 0.2 | 0.4 | 0.3 | 0.3±0.0 |
| **Mn** | 50 | n.a. | *0.1* | 0.7 | 0.4 | 0.2 | 0.1 | 0.4±0.1 |
| **Al** | 40 | n.a. | *5.3* | 17.5 | 2.7 | 5.1 | 2.9 | 7.1±3.5 |
| **Li** | n.a. | n.a. | *12.8* | 1.1 | 0.9 | 1.2 | 1.1 | 1.1±0.1 |
| **Cs** | n.a. | n.a. | <LOQ | <LOQ | <LOQ | <LOQ | <LOQ | 0.02±0.0 |
| **U** | 0.5 | 8.9 | ***0.6*** | <LOQ | <LOQ | <LOQ | <LOQ | 0.02±0.0 |
| **Co** | 0.6 | n.a. | *0.5* | <LOQ | <LOQ | <LOQ | <LOQ | 0.02±0.0 |
| **Sr** | 210 | n.a. | *790* | **307** | **286** | **338** | **286** | 304±12.3 |

^1^: Sample from Oct-13 excluded (see text).

| **Point 2 - Agricultural** | **AA-**  **EQS** | **MAC-EQS** | **Oct-13** | **Mar-14** | **Oct-14** | **Apr-15** | **Oct-15** | **Average** |
| --- | --- | --- | --- | --- | --- | --- | --- | --- |
| **Ba** | 73 | n.a. | **99** | **92** | **88** | **93** | **98** | **94**±2.1 |
| **Sb** | 5.6 | n.a. | 0.2 | 0.1 | 0.12 | 0.16 | <LOQ | 0.1±0.0 |
| **As** | 10 | n.a. | 1.5 | 0.4 | 3.1 | 2.9 | 2.7 | 2.1±0.5 |
| **Cd** | 0.2 | 0.45 | <LOQ | <LOQ | <LOQ | <LOQ | <LOQ | 0.02±0.0 |
| **Cr** | 2.5 | 7 | 0.3 | 0.3 | 0.3 | 0.3 | 0.3 | 0.3±0.0 |
| **Cu** | 7.8 | n.a. | 4.5 | 1.1 | 1.2 | 0.5 | 0.9 | 1.6±0.7 |
| **Pb** | 1.2 | 14 | 0.2 | 0.2 | 0.2 | 0.2 | 0.3 | 0.2±0.0 |
| **Hg** | 0.05 | 0.07 | <LOQ | <LOQ | <LOQ | <LOQ | <LOQ | 0.02±0.0 |
| **Ni** | 4 | 34 | 2.3 | 1.3 | 1.3 | 1.3 | 1.2 | 1.5±0.2 |
| **Se** | 1 | 10 | 0.8 | 0.9 | 0.5 | 0.3 | 0.2 | 0.5±0.1 |
| **V** | 3.5 | 50 | 2.1 | 1.2 | 2.6 | 2.7 | 2.5 | 2.2±0.3 |
| **Fe** | 200 | n.a. | 27.6 | 110 | 70.8 | 7.5 | 8.1 | 44.8±20.0 |
| **Zn** | 20.6 | n.a. | 6.9 | 3.1 | 4.6 | 5.5 | 0.6 | 4.1±1.1 |
| **Mn** | 50 | n.a. | 0.3 | 7.9 | 3.1 | 0.3 | 0.4 | 2.4±1.5 |
| **Al** | 40 | n.a. | **44.2** | **246** | **148** | 1.6 | 1.8 | **88.4**±47.7 |
| **Li** | n.a. | n.a. | 7.9 | 9.5 | 9.8 | 10.3 | 10.2 | 9.5±0.4 |
| **Cs** | n.a. | n.a. | <LOQ | 0.2 | 0.2 | 0.2 | 0.1 | 0.2±0.0 |
| **U** | 0.5 | 8.9 | **1.2** | **1.1** | **1.7** | **1.9** | **1.8** | 1.5±0.2 |
| **Co** | 0.6 | n.a. | 0.6 | 0.2 | 0.2 | 0.3 | 0.3 | 0.3±0.1 |
| **Sr** | 210 | n.a. | 672 | **783** | **879** | **861** | **805** | **800**±36.5 |

**Table SI2: Results for Metals (continued).**

| **Point 3 – Industrial/Urban** | **AA-**  **EQS** | **MAC-EQS** | **Oct-13** | **Mar-14** | **Oct-14** | **Apr-15** | **Oct-15** | **Average** |
| --- | --- | --- | --- | --- | --- | --- | --- | --- |
| **Ba** | 73 | n.a. | 50 | 48 | 46 | 42 | 44 | 45±1.3 |
| **Sb** | 5.6 | n.a. | 0.2 | 0.4 | 0.5 | 0.21 | <LOQ | 0.3±0.1 |
| **As** | 10 | n.a. | **58.3** | **34.2** | **64.1** | **62.6** | **56.2** | **54.3**±5.4 |
| **Cd** | 0.2 | 0.45 | <LOQ | <LOQ | <LOQ | <LOQ | <LOQ | 0.02±0.0 |
| **Cr** | 2.5 | 7 | 0.4 | 0.3 | 0.4 | 0.4 | 0.4 | 0.4±0.0 |
| **Cu** | 7.8 | n.a. | 1.1 | 0.6 | 0.8 | 0.4 | 1.5 | 0.8±0.2 |
| **Pb** | 1.2 | 14 | 0.2 | 0.4 | 0.4 | 0.2 | 0.2 | 0.3±0.0 |
| **Hg** | 0.05 | 0.07 | <LOQ | <LOQ | <LOQ | <LOQ | <LOQ | 0.02±0.0 |
| **Ni** | 4 | 34 | 1.9 | 0.7 | 1.1 | 1.2 | 1.6 | 1.2±0.2 |
| **Se** | 1 | 10 | 0.5 | 0.5 | 0.4 | 0.3 | 0.3 | 0.4±0.0 |
| **V** | 3.5 | 50 | **5.5** | **4.3** | **4.9** | **5.1** | **6.3** | **5.2**±0.3 |
| **Fe** | 200 | n.a. | 12.1 | 52.4 | 10.5 | 13.2 | 12.6 | 22.2±8.1 |
| **Zn** | 20.6 | n.a. | 10.2 | 7.2 | 6.1 | 2.4 | **28.5** | **11.1**±4.6 |
| **Mn** | 50 | n.a. | 0.2 | 25.6 | 0.6 | 0.2 | 1.1 | 6.9±5.0 |
| **Al** | 40 | n.a. | 3.9 | **61.5** | 0.7 | 1.4 | 2.1 | 16.4±11.9 |
| **Li** | n.a. | n.a. | 31.7 | 18.2 | 30.5 | 31.2 | 31.4 | **27.8**±2.6 |
| **Cs** | n.a. | n.a. | 6.3 | 3.1 | 6.5 | 6.6 | 4.3 | **5.1**±0.7 |
| **U** | 0.5 | 8.9 | **2.1** | **1.6** | **1.8** | **1.9** | **2.4** | **1.9**±0.1 |
| **Co** | 0.6 | n.a. | **1.6** | **0.7** | 0.4 | **0.9** | **0.9** | 0.7±0.2 |
| **Sr** | 210 | n.a. | 2435 | **1507** | **2512** | **2352** | **1885** | **2138**±191.8 |

| **Point 4 - Urban** | **AA-**  **EQS** | **MAC-EQS** | **Oct-13** | **Mar-14** | **Oct-14** | **Apr-15** | **Oct-15** | **Average** |
| --- | --- | --- | --- | --- | --- | --- | --- | --- |
| **Ba** | 73 | n.a. | 68 | 70 | 56 | 59 | 65 | 63±2.6 |
| **Sb** | 5.6 | n.a. | 0.3 | 0.2 | 0.8 | 0.24 | <LOQ | 0.3±0.1 |
| **As** | 10 | n.a. | 6.7 | 7.1 | 7.5 | 8.1 | 8.7 | 7.9±0.4 |
| **Cd** | 0.2 | 0.45 | <LOQ | <LOQ | <LOQ | <LOQ | <LOQ | 0.02±0.0 |
| **Cr** | 2.5 | 7 | 0.4 | 0.3 | 0.3 | 0.4 | 0.7 | 0.4±0.1 |
| **Cu** | 7.8 | n.a. | 1.2 | 1.2 | 2.5 | 1.7 | 0.3 | 1.4±0.4 |
| **Pb** | 1.2 | 14 | 0.2 | 1.1 | 0.3 | 0.3 | 0.3 | 0.5±0.2 |
| **Hg** | 0.05 | 0.07 | <LOQ | <LOQ | <LOQ | <LOQ | <LOQ | 0.02±0.0 |
| **Ni** | 4 | 34 | **4.9** | 1.2 | 1.2 | 1.3 | 0.6 | 1.1±0.8 |
| **Se** | 1 | 10 | 0.5 | 0.6 | 0.4 | 0.3 | 0.3 | 0.4±0.1 |
| **V** | 3.5 | 50 | 3.1 | 2.5 | 2.4 | 2.7 | 3.5 | 2.8±0.2 |
| **Fe** | 200 | n.a. | 10.2 | 81 | 9.2 | 7.8 | 22.3 | 30.1±14.0 |
| **Zn** | 20.6 | n.a. | 3.5 | 5.2 | 3.2 | 2.1 | 0.7 | 2.8±0.7 |
| **Mn** | 50 | n.a. | 0.2 | 35.1 | 0.7 | 0.4 | 0.4 | 9.2±6.9 |
| **Al** | 40 | n.a. | 3.8 | **96.2** | 2.1 | 1.5 | 0.9 | 25.2±18.8 |
| **Li** | n.a. | n.a. | 16.1 | 13.7 | 19.3 | 19.8 | 37.5 | **22.6**±4.2 |
| **Cs** | n.a. | n.a. | 0.5 | 0.4 | 0.3 | 0.6 | 0.5 | 0.5±0.1 |
| **U** | 0.5 | 8.9 | **1.4** | **1.4** | **1.2** | **1.5** | **1.7** | 1.5±0.1 |
| **Co** | 0.6 | n.a. | **0.7** | 0.4 | 0.5 | 0.6 | 0.3 | 0.5±0.1 |
| **Sr** | 210 | n.a. | 1187 | **1071** | **1377** | **1323** | **2096** | **1411**±179.4 |

**Table SI3: Inorganic ions and DOC.** Concentrations in mg/L.

Values in red highlight higher or lower than average temporal concentrations (within a sampling point; explained in the text). Values in **orange** highlight higher or lower than average spatial concentrations (between sampling points). Average of sampling campaigns ± standard error is reported in the last column.

| **Point 1 - Pristine** | ***Oct-13*** | **Mar-14** | **Oct-14** | **Apr-15** | **Oct-15** | **Average^1^** |
| --- | --- | --- | --- | --- | --- | --- |
| **NO_2_^-^** | *0.02* | < 0.008 | < 0.008 | < 0.008 | 0.01 | 0.01 |
| **NO_3_^-^** | *3.1* | 3.1 | 1.8 | 2.1 | 2.7 | 2.4±0.29 |
| **Cl^-^** | *12.3* | 4.9 | 16.3 | 16.8 | 16.1 | 13.5±2.88 |
| **SO_4_^2-^** | *57.1* | 10.8 | 7.8 | 7.9 | 7.1 | **8.4**±0.82 |
| **F^-^** | *0.16* | < 0.07 | < 0.07 | 0.10 | 0.10 | **0.05**±0.03 |
| **Ca^2+^** | *79.8* | 46.5 | 48.1 | 52.3 | 48.6 | 48.9±1.23 |
| **Mg^2+^** | *19.4* | 1.5 | 1.2 | 2.7 | 2.5 | 2.0±0.37 |
| **Na^+^** | *19.2* | 3.4 | 3.3 | 4.9 | 4.8 | 4.1±0.43 |
| **K^+^** | *3.7* | 0.4 | 0.4 | 0.4 | 0.6 | **0.5**±0.05 |
| **B^+^** | *0.090* | 0.012 | 0.028 | 0.018 | 0.012 | **0.018**±0.00 |
| **DOC** | *1.28* | 0.80 | 0.70 | 0.36 | 1.03 | 0.72±0.14 |

^1^: Sample from Oct-13 excluded (see text).

| **Point 2 - Agricultural** | **Oct-13** | **Mar-14** | **Oct-14** | **Apr-15** | **Oct-15** | **Average** |
| --- | --- | --- | --- | --- | --- | --- |
| **NO_2_^-^** | 0.03 | 0.02 | 0.02 | 0.02 | 0.03 | 0.02±0.00 |
| **NO_3_^-^** | 11.9 | 8.6 | 3.8 | 5.2 | 3.5 | 6.6±1.60 |
| **Cl^-^** | 19.7 | 18.2 | 28.5 | 28.1 | 31.3 | 25.2±2.61 |
| **SO_4_^2-^** | 59.3 | 58.2 | 66.4 | 71.2 | 62.9 | 63.6±2.38 |
| **F^-^** | 0.29 | 0.30 | 0.30 | 0.40 | 0.40 | 0.34±0.03 |
| **Ca^2+^** | 80.2 | 83.5 | 74.2 | 77.1 | 71.3 | 77. 3±2.15 |
| **Mg^2+^** | 15.1 | 17.4 | 15.5 | 17.2 | 17.5 | 16.5±0.51 |
| **Na^+^** | 20.5 | 18.2 | 20.7 | 24.1 | 27.2 | 22.1±1.58 |
| **K^+^** | 5.8 | 3.1 | 10.1 | 11.7 | 9.8 | 8.1±1.58 |
| **B^+^** | 0.080 | 0.070 | 0.090 | 0.107 | 0.095 | 0.088±0.01 |
| **DOC** | 2.64 | 1.94 | 0.90 | 1.94 | 1.62 | 1.81±0.28 |

| **Point 3 -Industrial/Urban** | **Oct-13** | **Mar-14** | **Oct-14** | **Apr-15** | **Oct-15** | **Average** |
| --- | --- | --- | --- | --- | --- | --- |
| **NO_2_^-^** | 0.05 | 0.05 | 0.07 | 0.06 | 0.05 | 0.06±0.00 |
| **NO_3_^-^** | 9.5 | 5.8 | 5.4 | 8.8 | 10.4 | 8.0±1.01 |
| **Cl^-^** | 65.5 | 34.6 | 59.8 | 104 | 52.6 | 63.3±11.43 |
| **SO_4_^2-^** | 242 | 238 | 223 | 245 | 165 | **223**±14.89 |
| **F^-^** | 0.57 | 0.50 | 0.50 | 0.60 | 0.60 | 0.55±0.02 |
| **Ca^2+^** | 216 | 154 | 120 | 184 | 178 | **170**±15.95 |
| **Mg^2+^** | 46.2 | 30.1 | 36.3 | 42.8 | 35.9 | **38.3**±2.83 |
| **Na^+^** | 55.2 | 31.7 | 46.1 | 51.2 | 48.3 | **46.5**±4.00 |
| **K^+^** | 13.5 | 8.3 | 11.9 | 13.3 | 13.9 | 12.2±1.03 |
| **B^+^** | 0.650 | 0.390 | 0.460 | 0.654 | 0.542 | **0.539**±0.05 |
| **DOC** | 1.06 | 1.95 | 1.90 | 0.97 | 24.2 | 6.02±4.55 |

| **Point 4 - Urban** | **Oct-13** | **Mar-14** | **Oct-14** | **Apr-15** | **Oct-15** | **Average** |
| --- | --- | --- | --- | --- | --- | --- |
| **NO_2_^-^** | 0.04 | 0.04 | 0.06 | 0.08 | 0.09 | 0.06±0.01 |
| **NO_3_^-^** | 11.2 | 7.5 | 6.7 | 9.1 | 12.1 | 9.3±1.04 |
| **Cl^-^** | 157 | 78.5 | 348 | 352 | 514 | **290**±77.40 |
| **SO_4_^2-^** | 123 | 118 | 178 | 181 | 331 | **186**±38.54 |
| **F^-^** | 0.39 | 0.40 | 0.50 | 0.60 | 0.80 | 0.54±0.08 |
| **Ca^2+^** | 121 | 109 | 203 | 124 | 168 | **145**±17.61 |
| **Mg^2+^** | 28.2 | 23.2 | 48.2 | 49.4 | 111 | **51.9**±15.57 |
| **Na^+^** | 106 | 62.8 | 261 | 282 | 773 | **297**±126.32 |
| **K^+^** | 8.5 | 6.5 | 14.1 | 15.4 | 38.6 | 16.6±5.74 |
| **B^+^** | 0.180 | 0.170 | 0.190 | 0.285 | 0.506 | **0.266**±0.06 |
| **DOC** | 1.82 | 1.58 | 1.60 | 6.04 | 40.2 | 10.25±7.54 |

**Table SI4: PAHs results.** All concentrations in ng/L. LOQ: Limit of Quantification; AA-EQS = annual average EQS; MAC-EQS = maximum allowable concentration EQS.

Values in red highlight higher or lower than average temporal concentrations (within a sampling point; explained in the text). Values in **dark red bold**: EQS exceedance. Values < LOQ were considered half of the LOQ for average calculation. Average of sampling campaigns ± standard error is reported in the last column.

| **Point 1 - Pristine** | **LOQ** | **AA-EQS** | **MAC-EQS** | ***Oct-13*** | **Mar-14** | **Oct-14** | **Apr-15** | **Oct-15** | **Average^1^** |
| --- | --- | --- | --- | --- | --- | --- | --- | --- | --- |
| **Naphthalene** | 1.2 | 2400 | 130000 | *14.9* | < 1.2 | < 1.2 | 3.3 | 3.2 | 1.9±0.8 |
| **Acenaphthene** | 0.09 | 1300 |  | *0.23* | < 0.09 | 0.63 | 0.17 | 0.36 | 0.30±0.1 |
| **Fluorene** | 0.1 | 1500 |  | *0.27* | < 0.1 | < 0.1 | < 0.1 | 0.23 | 0.10±0.0 |
| **Phenanthrene** | 0.2 | 300 |  | *8.4* | < 0.2 | 1.7 | 1.0 | 1.9 | 1.2±0.4 |
| **Anthracene** | 0.1 | 100 | 100 | *< 0.1* | < 0.1 | < 0.1 | 0.35 | < 0.1 | 0.13±0.1 |
| **Fluoranthene** | 0.2 | 6.3 | 120 | *3.8* | < 0.2 | 0.35 | 0.31 | 1.8 | 0.6±0.4 |
| **Pyrene** | 0.1 | 23 |  | *9.0* | < 0.1 | < 0.1 | 0.16 | 0.49 | 0.19±0.1 |
| **Benzo(a)anthracene** | 0.05 | 0.23 |  | ***0.39*** | < 0.05 | < 0.05 | 0.06 | 0.17 | 0.07±0.0 |
| **Chrysene** | 0.2 | 1.2 |  | ***1.7*** | < 0.2 | < 0.2 | 0.29 | 0.79 | 0.32±0.2 |
| **Benzo(b)fluoranthene** | 0.01 | 30 | 17 | *< 0.01* | < 0.01 | < 0.01 | 0.11 | 0.12 | 0.06±0.0 |
| **Benzo(k)fluoranthene** | 0.02 | 30 | 17 | *< 0.02* | < 0.02 | < 0.02 | 0.05 | 0.08 | 0.04±0.0 |
| **Benzo(a)pyrene** | 0.01 | 0.17 | 270 | *< 0.01* | < 0.01 | < 0.01 | < 0.01 | 0.03 | 0.01±0.0 |
| **Dibenzo(a,h)anthracene** | 0.1 | 1.4 |  | *< 0.1* | < 0.1 | < 0.1 | < 0.1 | 0.29 | 0.11±0.1 |
| **Benzo(g,h,i)perylene** | 0.04 | 2 | 8.2 | *< 0.04* | < 0.04 | < 0.04 | < 0.04 | 0.05 | 0.03±0.0 |
| **Indeno(1,2,3-cd)pyrene** | 0.05 | 2 |  | *< 0.05* | < 0.05 | < 0.05 | < 0.05 | 0.08 | 0.04±0.0 |
|  |  |  |  |  |  |  |  | **Sum** | 5.1 |

^1^: Sample from Oct-13 excluded (see text).

| **Point 2 - Agricultural** | **LOQ** | **AA-EQS** | **MAC-EQS** | **Oct-13** | **Mar-14** | **Oct-14** | **Apr-15** | **Oct-15** | **Average** |
| --- | --- | --- | --- | --- | --- | --- | --- | --- | --- |
| **Naphthalene** | 1.2 | 2400 | 130000 | 9.7 | 6.5 | 10.1 | 44.2 | 4.8 | 15.1±7.3 |
| **Acenaphthene** | 0.09 | 1300 |  | 2.1 | 0.95 | 1.6 | 1.7 | 0.70 | 1.4±0.3 |
| **Fluorene** | 0.1 | 1500 |  | 2.1 | 1.2 | 1.7 | 1.4 | 0.72 | 1.4±0.2 |
| **Phenanthrene** | 0.2 | 300 |  | 2.7 | 2.1 | 10.9 | 44.0 | 37.8 | 19.5±8.9 |
| **Anthracene** | 0.1 | 100 | 100 | 4.7 | 3.5 | < 0.1 | 1.3 | 2.1 | 2.3±0.8 |
| **Fluoranthene** | 0.2 | 6.3 | 120 | < 0.2 | 2.3 | 2.6 | 4.8 | 2.8 | 2.5±0.8 |
| **Pyrene** | 0.1 | 23 |  | 9.5 | 8.6 | 1.7 | 1.6 | 0.97 | 4.5±1.9 |
| **Benzo(a)anthracene** | 0.05 | 0.23 |  | < 0.05 | < 0.05 | < 0.05 | **0.36** | **0.48** | 0.18±0.1 |
| **Chrysene** | 0.2 | 1.2 |  | < 0.2 | < 0.2 | < 0.2 | **2.0** | **2.0** | 0.9±0.5 |
| **Benzo(b)fluoranthene** | 0.01 | 30 | 17 | 1.0 | 1.2 | 5.2 | 0.90 | 1.0 | 1.9±0.8 |
| **Benzo(k)fluoranthene** | 0.02 | 30 | 17 | 0.17 | 0.9 | < 0.02 | 4.3 | 1.5 | 1.4±0.8 |
| **Benzo(a)pyrene** | 0.01 | 0.17 | 270 | 0.01 | 0.1 | < 0.01 | 0.03 | 0.02 | 0.03±0.0 |
| **Dibenzo(a,h)anthracene** | 0.1 | 1.4 |  | 0.28 | < 0.1 | < 0.1 | 0.50 | 0.59 | 0.30±0.1 |
| **Benzo(g,h,i)perylene** | 0.04 | *2* | 8.2 | < 0.04 | < 0.04 | < 0.04 | 1.1 | 1.2 | 0.5±0.3 |
| **Indeno(1,2,3-cd)pyrene** | 0.05 | *2* |  | < 0.05 | < 0.05 | < 0.05 | 0.37 | 0.56 | 0.20±0.1 |
|  |  |  |  |  |  |  |  | **Sum** | 52.0 |

**Table SI4: PAHs results (continued).**

| **Point 3 – Industrial/ Urban** | **LOQ** | **AA-EQS** | **MAC-EQS** | **Oct-13** | **Mar-14** | **Oct-14** | **Apr-15** | **Oct-15** | **Average** |
| --- | --- | --- | --- | --- | --- | --- | --- | --- | --- |
| **Naphthalene** | 1.2 | 2400 | 130000 | 62.0 | 51.3 | 23.7 | 31.5 | 32.8 | 40.3±7.1 |
| **Acenaphthene** | 0.09 | 1300 |  | 2.0 | 2.1 | 1.3 | 0.10 | 0.43 | 1.2±0.4 |
| **Fluorene** | 0.1 | 1500 |  | 2.0 | 2.6 | 1.69 | 0.20 | 0.80 | 1.5±0.4 |
| **Phenanthrene** | 0.2 | 300 |  | 4.7 | 3.6 | 4.8 | 16.1 | 8.9 | 7.6±2.3 |
| **Anthracene** | 0.1 | 100 | 100 | 5.7 | 4.2 | 3.4 | 0.10 | 3.1 | 3.3±0.9 |
| **Fluoranthene** | 0.2 | 6.3 | 120 | 2.4 | 1.1 | 1.1 | 0.91 | 1.2 | 1.3±0.3 |
| **Pyrene** | 0.1 | 23 |  | 12.1 | 9.3 | 8.8 | 0.31 | 0.24 | 6.2±2.5 |
| **Benzo(a)anthracene** | 0.05 | 0.23 |  | < 0.05 | < 0.05 | < 0.05 | 0.09 | 0.06 | 0.04±0.0 |
| **Chrysene** | 0.2 | 1.2 |  | < 0.2 | < 0.2 | < 0.2 | 0.31 | 0.24 | 0.17±0.0 |
| **Benzo(b)fluoranthene** | 0.01 | 30 | 17 | 0.43 | 0.9 | 3.7 | 0.27 | 0.06 | 1.1±0.7 |
| **Benzo(k)fluoranthene** | 0.02 | 30 | 17 | 0.12 | 0.2 | < 0.02 | 1.6 | 0.46 | 0.49±0.3 |
| **Benzo(a)pyrene** | 0.01 | 0.17 | 270 | < 0.01 | **0.9** | < 0.01 | 0.01 | 0.04 | 0.19±0.2 |
| **Dibenzo(a,h)anthracene** | 0.1 | 1.4 |  | < 0.1 | < 0.1 | < 0.1 | 0.16 | 0.15 | 0.09±0.0 |
| **Benzo(g,h,i)perylene** | 0.04 | *2* | 8.2 | < 0.04 | < 0.04 | < 0.04 | 0.30 | 0.25 | 0.12±0.1 |
| **Indeno(1,2,3-cd)pyrene** | 0.05 | *2* |  | < 0.05 | < 0.05 | < 0.05 | 0.81 | 0.54 | 0.28±0.2 |
|  |  |  |  |  |  |  |  | **Sum** | 63.8 |

| **Point 4 - Urban** | **LOQ** | **AA-EQS** | **MAC-EQS** | **Oct-13** | **Mar-14** | **Oct-14** | **Apr-15** | **Oct-15** | **Average** |
| --- | --- | --- | --- | --- | --- | --- | --- | --- | --- |
| **Naphthalene** | 1.2 | 2400 | 130000 | 29.1 | 11.5 | 24.2 | 69.7 | 48.6 | 36.6±10.2 |
| **Acenaphthene** | 0.09 | 1300 |  | 0.89 | 0.98 | 0.75 | 0.75 | 1.4 | 1.0±0.1 |
| **Fluorene** | 0.1 | 1500 |  | 0.89 | 1.5 | 1.7 | 1.2 | 0.23 | 1.1±0.3 |
| **Phenanthrene** | 0.2 | 300 |  | 7.0 | 6.3 | 8.6 | 15.7 | 15.7 | 10.7±2.1 |
| **Anthracene** | 0.1 | 100 | 100 | 5.9 | 3.2 | 1.2 | < 0.1 | 0.72 | 2.2±1.1 |
| **Fluoranthene** | 0.2 | 6.3 | 120 | 2.0 | 1.3 | 3.9 | 2.4 | 3.0 | 2.5±0.5 |
| **Pyrene** | 0.1 | 23 |  | 15.3 | 11.2 | 4.1 | 0.92 | 0.95 | 6.5±2.9 |
| **Benzo(a)anthracene** | 0.05 | 0.23 |  | < 0.05 | < 0.05 | 0.27 | 0.12 | **0.37** | 0.16±0.1 |
| **Chrysene** | 0.2 | 1.2 |  | 0.67 | 0.9 | 0.35 | 0.34 | **1.6** | 0.78±0.2 |
| **Benzo(b)fluoranthene** | 0.01 | 30 | 17 | 1.6 | 1.4 | < 0.01 | 0.02 | 0.84 | 0.8±0.3 |
| **Benzo(k)fluoranthene** | 0.02 | 30 | 17 | 0.19 | 0.12 | 0.68 | 2.8 | 0.50 | 0.86±0.5 |
| **Benzo(a)pyrene** | 0.01 | 0.17 | 270 | 0.02 | **0.8** | 0.07 | 0.02 | 0.02 | 0.19±0.2 |
| **Dibenzo(a,h)anthracene** | 0.1 | 1.4 |  | 0.62 | 0.6 | < 0.1 | 0.16 | 0.49 | 0.38±0.1 |
| **Benzo(g,h,i)perylene** | 0.04 | 2 | 8.2 | 0.44 | 0.4 | < 0.04 | 0.20 | 1.2 | 0.46±0.2 |
| **Indeno(1,2,3-cd)pyrene** | 0.05 | 2 |  | < 0.05 | < 0.05 | < 0.05 | 1.4 | **2.8** | 0.8±0.5 |
|  |  |  |  |  |  |  |  | **Sum** | 65.0 |

**Table SI5: List of internal surrogate standards used for isotope dilution analysis.**

| 1-H-Benzotriazole d_4_ |
| --- |
| Carbamazepine d_10_ |
| ^13^C_6_-Diclofenac |
| ^13^C_6_-Sulfamethoxazole |
| Cybutryne d_9_ |
| DEET (N,N-diethyl-m-toluamide) d_6_ |
| 2,4-D (2,4-dichlorophenoxyacetic acid) d_6_ |
| MCPA (2-methyl-4-chlorophenoxyacetic acid) d_3_ |
| Metolachlor d_6_ |
| Terbutryn d_5_ |
| ^13^C_2_-PFHxA (perfluorohexanoic acid) |
| ^18^O_2_-PFHxS (perfluorohexane sulfonic acid) |
| ^13^C_4_-PFOA (perfluorooctanoic acid) |
| ^13^C_4_-PFOS (perfluorooctane sulfonic acid) |
| ^13^C_5_-PFNA (perfluorononanoic acid) |

**Table SI6: UHPLC-MS-MS results.** All concentrations in ng/L. EQS = Environmental Quality Standard.

**Values in red highlight higher or lower than average temporal concentrations (within a sampling point; explained in the text). Values in dark red bold: EQS exceedance. Values < LOQ were considered half of the LOQ for average calculation. Average of sampling campaigns ± standard error is reported in the last column. Con.: Sample was contaminated in the laboratory with PFAS. n.a.: not applicable, the chemical is not reported in any list with an EQS value (see paragraph 2.6 of the main text).**

| **Point 1 - Pristine** | **EQS** | **LOQ** | ***Oct-13*** | **Mar-14** | **Oct-14** | **Apr-15** | **Oct-15** | **Average^1^** |
| --- | --- | --- | --- | --- | --- | --- | --- | --- |
| **1H-Benzotriazole** | 30000 | 0.7 | *9.2* | 1.7 | 1.3 | 8.1 | 0.8 | 3.0±1.7 |
| **Methylbenzotriazoles** | 150000 | 0.5 | *11.4* | 2.1 | < 0.5 | < 0.5 | < 0.5 | 0.7±0.5 |
| **Carbamazepine (CBZ)** | 500 | 0.2 | *1.2* | < 0.2 | < 0.2 | < 0.2 | < 0.2 | 0.1±0.0 |
| **CBZ-diOH** | 500 | 0.3 | *8.0* | < 0.3 | < 0.3 | < 0.3 | < 0.3 | 0.2±0.0 |
| **Diclofenac** | 50 | 0.9 | *< 0.9* | 1.4 | < 0.9 | < 0.9 | < 0.9 | 0.7±0.2 |
| **Sulfamethoxazole** | 600 | 0.1 | *1.8* | < 0.1 | < 0.1 | < 0.1 | < 0.1 | 0.1±0.0 |
| **Cybutryne (Irgarol)** | 2.5 | 0.2 | *< 0.2* | < 0.2 | < 0.2 | < 0.2 | < 0.2 | 0.1±0.0 |
| **DEET** | 41000 | 1.9 | *< 1.9* | < 1.9 | < 1.9 | < 1.9 | 14.5 | 4.3±3.4 |
| **2,4-D** | 100 | 0.2 | *< 0.2* | < 0.2 | < 0.2 | < 0.2 | 1.8 | 0.5±0.4 |
| **MCPA** | 100 | 0.2 | *< 0.2* | < 0.2 | < 0.2 | < 0.2 | < 0.2 | 0.1±0.0 |
| **Metolachlor** | 200 | 1.7 | *< 1.7* | < 1.7 | < 1.7 | < 1.7 | < 1.7 | 0.9±0.0 |
| **Terbutryn** | 65 | 0.6 | *1.4* | 0.9 | < 0.6 | < 0.6 | < 0.6 | 0.5±0.2 |
| **Terbutylazine** | 220 | 0.5 | *< 0.5* | < 0.5 | < 0.5 | < 0.5 | < 0.5 | 0.3±0.0 |
| **PFBS** | 3000 | 0.6 | *6.0* | 15.2 | < 0.6 | < 0.6 | < 0.6 | 4.0±3.7 |
| **PFHxA** | 1000 | 1.1 | *< 1.1* | < 1.1 | < 1.1 | < 1.1 | 7.2 | 2.2±1.7 |
| **PFHpA** | n.a. | 3.2 | *< 3.2* | < 3.2 | < 3.2 | *Con.* | 4.3 | 2.5±0.8 |
| **PFOA** | 100 | 1.1 | *1.2* | 1.3 | 1.2 | *Con.* | 7.1 | 3.2±1.7 |
| **PFNA** | n.a. | 0.7 | *0.7* | < 0.7 | < 0.7 | *Con.* | < 0.7 | 0.4±0.0 |
| **PFOS** | 0.65 | 1.1 | *< 1.1* | < 1.1 | < 1.1 | < 1.1 | **1.7** | 0.8±0.3 |

^1^: Sample from Oct-13 excluded (see text).

| **Point 2 - Agricultural** | **EQS** | **LOQ** | **Oct-13** | **Mar-14** | **Oct-14** | **Apr-15** | **Oct-15** | **Average** |
| --- | --- | --- | --- | --- | --- | --- | --- | --- |
| **1H-Benzotriazole** | 30000 | 0.7 | 91.4 | 44.8 | 91.1 | 46.4 | 142 | 83.1±17.9 |
| **Methylbenzotriazoles** | 150000 | 0.5 | 563 | 103 | 224 | 216 | 148 | 251±81.2 |
| **Carbamazepine (CBZ)** | 500 | 0.2 | 8.7 | 3.8 | 10.4 | 3.7 | 12.4 | 7.8±1.8 |
| **CBZ-diOH** | 500 | 0.3 | 84.0 | 28.3 | 78.0 | 50.7 | 148 | 77.8±20.2 |
| **Diclofenac** | 50 | 0.9 | 26.5 | 9.1 | 10.5 | 8.0 | 7.7 | 12.4±3.6 |
| **Sulfamethoxazole** | 600 | 0.1 | 15.6 | 8.8 | 13.2 | 5.5 | 18.2 | 12.3±2.3 |
| **Cybutryne (Irgarol)** | 2.5 | 0.2 | < 0.2 | < 0.2 | < 0.2 | < 0.2 | < 0.2 | 0.1±0.0 |
| **DEET** | 41000 | 1.9 | 26.6 | 2.5 | 33.0 | 2.0 | 21.7 | 17.2±6.3 |
| **2,4-D** | 100 | 0.2 | 27.0 | 2.5 | 2.5 | 2.3 | 2.0 | 7.3±4.9 |
| **MCPA** | 100 | 0.2 | 30.4 | 8.2 | 2.4 | 19.1 | 1.2 | 12.2±5.5 |
| **Metolachlor** | 200 | 1.7 | 31.6 | 3.7 | 5.1 | 15.7 | 8.3 | 12.9±5.1 |
| **Terbutryn** | 65 | 0.6 | 3.5 | 1.2 | 1.3 | < 0.6 | 1.7 | 1.6±0.5 |
| **Terbutylazine** | 220 | 0.5 | 12.7 | 3.0 | 3.1 | 11.3 | 9.6 | 7.9±2.1 |
| **PFBS** | 3000 | 0.6 | < 0.6 | 3.6 | < 0.6 | < 0.6 | < 0.6 | 1.0±0.7 |
| **PFHxA** | 1000 | 1.1 | 1.6 | 1.2 | 1.3 | 1.5 | 3.8 | 1.9±0.5 |
| **PFHpA** | n.a. | 3.2 | < 3.2 | < 3.2 | < 3.2 | *Con.* | < 3.2 | 1.6±0.0 |
| **PFOA** | 100 | 1.1 | 3.6 | 1.3 | 3.7 | *Con.* | 14.1 | 5.7±2.6 |
| **PFNA** | n.a. | 0.7 | 2.6 | < 0.7 | < 0.7 | *Con.* | 2.8 | 1.5±0.6 |
| **PFOS** | 0.65 | 1.1 | **2.9** | **1.2** | 1.1 | < 1.1 | 1.1 | 1.4±0.4 |

**Table SI6: UHPLC-MS-MS results (continued).**

| **Point 3 – Industrial / Urban** | **EQS** | **LOQ** | **Oct-13** | **Mar-14** | **Oct-14** | **Apr-15** | **Oct-15** | **Average** |
| --- | --- | --- | --- | --- | --- | --- | --- | --- |
| **1H-Benzotriazole** | 30000 | 0.7 | 317 | 187 | 356 | 180 | 516 | 311±61.9 |
| **Methylbenzotriazoles** | 150000 | 0.5 | 297 | 338 | 310 | 335 | 347 | 326±9.4 |
| **Carbamazepine (CBZ)** | 500 | 0.2 | 21.9 | 9.5 | 23.0 | 14.6 | 14.1 | 16.6±2.5 |
| **CBZ-diOH** | 500 | 0.3 | 200 | 86.2 | 201 | 176 | 225 | 178±24.1 |
| **Diclofenac** | 50 | 0.9 | 119 | 49.0 | **159** | **103** | **849** | 256±149.3 |
| **Sulfamethoxazole** | 600 | 0.1 | 32.9 | 25.5 | 33.6 | 34.3 | 63.0 | 37.9±6.5 |
| **Cybutryne (Irgarol)** | 2.5 | 0.2 | < 0.2 | < 0.2 | < 0.2 | < 0.2 | < 0.2 | 0.1±0.0 |
| **DEET** | 41000 | 1.9 | 40.0 | 6.0 | 34.0 | 3.6 | 44.4 | 25.6±8.7 |
| **2,4-D** | 100 | 0.2 | 0.9 | 10.5 | 0.9 | 0.8 | 0.5 | 2.7±1.9 |
| **MCPA** | 100 | 0.2 | < 0.2 | 5.7 | < 0.2 | < 0.2 | 0.2 | 1.2±1.1 |
| **Metolachlor** | 200 | 1.7 | < 1.7 | 10.8 | < 1.7 | < 1.7 | < 1.7 | 2.8±2.0 |
| **Terbutryn** | 65 | 0.6 | 9.3 | 3.5 | 3.1 | 2.2 | 4.8 | 4.6±1.2 |
| **Terbutylazine** | 220 | 0.5 | < 0.5 | 6.7 | < 0.5 | < 0.5 | < 0.5 | 1.5±1.3 |
| **PFBS** | 3000 | 0.6 | 10.8 | 9.2 | < 0.6 | < 0.6 | < 0.6 | 4.2±2.4 |
| **PFHxA** | 1000 | 1.1 | 1.4 | 3.1 | 1.5 | < 1.1 | < 1.1 | 1.4±0.5 |
| **PFHpA** | n.a. | 3.2 | < 3.2 | < 3.2 | < 3.2 | *Con.* | 16.9 | 5.4±3.4 |
| **PFOA** | 100 | 1.1 | 9.5 | 4.9 | 3.6 | *Con.* | 33.4 | 12.8±6.2 |
| **PFNA** | n.a. | 0.7 | < 0.7 | < 0.7 | 2.6 | *Con.* | 10.6 | 3.5±2.2 |
| **PFOS** | 0.65 | 1.1 | **2.5** | **13.8** | < 1.1 | < 1.1 | < 1.1 | 3.6±2.6 |

| **Point 4 - Urban** | **EQS** | **LOQ** | **Oct-13** | **Mar-14** | **Oct-14** | **Apr-15** | **Oct-15** | **Average** |
| --- | --- | --- | --- | --- | --- | --- | --- | --- |
| **1H-Benzotriazole** | 30000 | 0.7 | 261 | 54.5 | 380 | 186 | 425 | 261±66.8 |
| **Methylbenzotriazoles** | 150000 | 0.5 | 570 | 852 | 318 | 536 | 555 | 566±84.9 |
| **Carbamazepine (CBZ)** | 500 | 0.2 | 12.0 | 46.0 | 12.9 | 9.1 | 16.2 | 19.2±6.8 |
| **CBZ-diOH** | 500 | 0.3 | 153 | 51.8 | 123 | 126 | 236 | 138±29.7 |
| **Diclofenac** | 50 | 0.9 | **62.4** | 42.1 | **100** | **69.0** | **158** | 86.3±20.2 |
| **Sulfamethoxazole** | 600 | 0.1 | 29.3 | 59.0 | 35.0 | 32.4 | 79.0 | 46.9±9.6 |
| **Cybutryne (Irgarol)** | 2.5 | 0.2 | **2.9** | **4.5** | 0.9 | < 0.2 | < 0.2 | 1.7±0.9 |
| **DEET** | 41000 | 1.9 | 66.2 | 42.1 | 45.0 | 4.8 | 30.4 | 37.7±10.0 |
| **2,4-D** | 100 | 0.2 | 11.2 | 46.6 | 2.0 | 2.0 | 1.8 | 12.7±8.7 |
| **MCPA** | 100 | 0.2 | 1.8 | 54.6 | 1.6 | 7.0 | 0.3 | 13.1±10.4 |
| **Metolachlor** | 200 | 1.7 | 5.0 | 53.0 | < 1.7 | 6.3 | < 1.7 | 13.2±10.0 |
| **Terbutryn** | 65 | 0.6 | 6.6 | 46.3 | 1.9 | 1.8 | 1.1 | 11.5±8.8 |
| **Terbutylazine** | 220 | 0.5 | 5.4 | 44.0 | < 0.5 | 5.9 | 1.0 | 11.3±8.2 |
| **PFBS** | 3000 | 0.6 | 6.0 | 22.1 | < 0.6 | < 0.6 | 2.1 | 6.2±4.1 |
| **PFHxA** | 1000 | 1.1 | 3.2 | 23.7 | 2.6 | 2.6 | < 1.1 | 6.5±4.3 |
| **PFHpA** | n.a. | 3.2 | < 3.2 | 26.6 | < 3.2 | *Con.* | < 3.2 | 9.9±6.4 |
| **PFOA** | 100 | 1.1 | 6.5 | 23.6 | 2.9 | *Con.* | < 1.1 | 8.4±4.7 |
| **PFNA** | n.a. | 0.7 | 1.0 | 20.7 | < 0.7 | *Con.* | 1.2 | 5.8±4.4 |
| **PFOS** | 0.65 | 1.1 | **2.3** | **24.0** | < 1.1 | < 1.1 | < 1.1 | 5.6±4.6 |

**Table SI7: Results of the microbiological analyses. Average of sampling campaigns ± standard error is reported in the last column.**

|  | **Point 1 - Pristine** | | | | | |
| --- | --- | --- | --- | --- | --- | --- |
|  | ***Oct-13*** | **Mar-14** | **Oct-14** | **Apr-15** | **Oct-15** | **Average^1^** |
| **Microbial abundance (cells/mL)** | *5.6x10^5^* | 1.0x10^5^ | 5.4x10^5^ | 1.7x10^5^ | 1.4x10^5^ | 2.4x10^5^±1.0x10^5^ |
| **Viability (% live cells)** | *7.1* | 67.0 | 41.5 | 77.3 | 77.6 | 65.8±8.5 |
| ***Bacteria* (% positive cells vs DAPI)** | *32.3* | 62.3 | 32.9 | 13.2 | 34.6 | 35.7±10.1 |
| ***Archaea* (% positive cells vs DAPI)** | *2.0* | 0.2 | 1.0 | 0.0 | 0.0 | 0.3±0.2 |
| **Total Coliforms (MPN/100 mL)** | *1568* | 55 | <1 | 1 | 55 | 27.9±15.7 |
| ***E. coli* (MPN/100 mL)** | *40* | 19 | <1 | <1 | 24 | 11.0±6.1 |
| ***Enterococcus* spp. (MPN/100 mL)** | *20* | <1 | 20 | <1 | 62 | 20.8±14.5 |
| **^1^: Sample from Oct-13 excluded (see text)** | | | | | | |
|  | **Point 2 - Agricultural** | | | | | |
|  | **Oct-13** | **Mar-14** | **Oct-14** | **Apr-15** | **Oct-15** | **Average** |
| **Microbial abundance (cells/mL)** | 1.8x10^6^ | 3.3x10^6^ | 6.8x10^5^ | 7.3x10^5^ | 8.2x10^5^ | 1.5x10^6^±4.9x10^5^ |
| **Viability (% live cells)** | 51.4 | 62.8 | 75.7 | 68.6 | 87.9 | 69.3±6.1 |
| ***Bacteria* (% positive cells vs DAPI)** | 48.3 | 32.8 | 36.8 | 11.9 | 39.2 | 33.8±6.0 |
| ***Archaea* (% positive cells vs DAPI)** | 1.8 | 0.5 | 0.3 | 0.0 | 0.6 | 0.6±0.3 |
| **Total Coliforms (MPN/100 mL)** | 2708 | 6817 | 21 | 308 | 1534 | 2278±1231.2 |
| ***E. coli* (MPN/100 mL)** | 610 | 470 | 16 | 96 | 167 | 272±114.2 |
| ***Enterococcus* spp. (MPN/100 mL)** | 62 | 20 | 291 | 4 | 377 | 151±76.6 |

**Table SI7: Results of the microbiological analyses (continued)**

|  | **Point 3 – Industrial/Urban** | | | | | |
| --- | --- | --- | --- | --- | --- | --- |
|  | **Oct-13** | **Mar-14** | **Oct-14** | **Apr-15** | **Oct-15** | **Average** |
| **Microbial abundance (cells/mL)** | 1.9x10^6^ | 8.9x10^5^ | 8.0x10^5^ | 6.2x10^5^ | 2.8x10^6^ | 1.4x10^6^±4.1x10^5^ |
| **Viability (% live cells)** | 79.7 | 66.5 | 67.0 | 91.5 | 76.1 | 76.1±4.6 |
| ***Bacteria* (% positive cells vs DAPI)** | 62.3 | 67.8 | 73.0 | 41.2 | 64.6 | 61.8±5.4 |
| ***Archaea* (% positive cells vs DAPI)** | 1.0 | 0.2 | 0.1 | 0.3 | 0.7 | 0.5±0.2 |
| **Total Coliforms (MPN/100 mL)** | 856 | >48392 | 223 | >2419 | 83270 | >27032±16776.0 |
| ***E. coli* (MPN/100 mL)** | 62 | 8396 | 223 | >2419 | 6950 | >3610±1725.4 |
| ***Enterococcus* spp. (MPN/100 mL)** | 20 | 3483 | 1553 | >2419 | 6825 | >2860±1141.9 |
|  | **Point 4 - Urban** | | | | | |
|  | **Oct-13** | **Mar-14** | **Oct-14** | **Apr-15** | **Oct-15** | **Average** |
| **Microbial abundance (cells/mL)** | 2.7x10^6^ | 1.4x10^6^ | 1.1x10^6^ | 4.8x10^5^ | 2.2x10^6^ | 1.6x10^6^±4.0x10^5^ |
| **Viability (% live cells)** | 7.6 | 67.1 | 62.6 | 57.9 | 80.9 | 55.2±12.5 |
| ***Bacteria* (% positive cells vs DAPI)** | 37.4 | 48.7 | 58.3 | 25.1 | 37.0 | 41.3±5.7 |
| ***Archaea* (% positive cells vs DAPI)** | 0.2 | 0.0 | 0.0 | 0.5 | 0.3 | 0.2±0.1 |
| **Total Coliforms (MPN/100 mL)** | 1096 | >48392 | 1011 | >2419 | 8880 | >12360±9123.9 |
| ***E. coli* (MPN/100 mL)** | 150 | 17001 | 1011 | >2419 | 1945 | >4505±3148.2 |
| ***Enterococcus* spp. (MPN/100 mL)** | <1 | 4617 | >2419 | >2419 | 310 | >2464±837.9 |

Note: for cases where counts were not obtained, the limit of detection was below 5 cells in 30 fields.

**Table SI8: Bacterial community phylogenetic composition, analyzed by FISH, expressed in cells/mL. α-, β-, γ-, δ-, ε-: *Proteobacteria*; Pla: *Planctomycetes*; CF: *Cytophaga-Flavobacterium lineage of the Bacteroidetes*. Average of sampling campaigns ± standard error is reported in the last column.**

|  | **Point 1 - Pristine** | | | | |  |
| --- | --- | --- | --- | --- | --- | --- |
|  | ***Oct-13*** | **Mar-14** | **Oct-14** | **Apr-15** | **Oct-15** | **Average^1^** |
| **α-** | *2.19x10^4^* | 2.00x10^3^ | 8.21x10^3^ | 2.52x10^3^ | 2.91x10^3^ | 3.91x10^3^±1.4x10^3^ |
| **β-** | *6.85x10^4^* | 2.54x10^4^ | 4.30x10^4^ | 3.60x10^3^ | 5.79x10^3^ | 1.94x10^4^±9.3x10^3^ |
| **γ-** | *1.39x10^4^* | 3.68x10^3^ | 2.10x10^4^ | 2.78x10^3^ | 5.53x10^3^ | 8.26x10^3^±4.3x10^3^ |
| **δ-** | *2.13x10^4^* | 3.70x10^3^ | 2.15x10^4^ | 5.37x10^2^ | 0.00 | 6.45x10^3^±5.1x10^3^ |
| **ε-** | *1.81x10^3^* | 0.00 | 0.00 | 1.68x10^3^ | 0.00 | 4.21x10^2^±4.2x10^2^ |
| **Pla** | *0.00* | 1.70x10^2^ | 2.62x10^3^ | 1.37x10^3^ | 2.82x10^3^ | 1.75x10^3^±6.2x10^2^ |
| **CF** | *0.00* | 6.93x10^3^ | 0.00 | 2.39x10^3^ | 1.60x10^3^ | 2.73x10^3^±1.5x10^3^ |
|  | **Point 2 - Agricultural** | | | | |  |
|  | **Oct-13** | **Mar-14** | **Oct-14** | **Apr-15** | **Oct-15** | **Average** |
| **α-** | 4.64x10^4^ | 2.65x10^4^ | 1.08x10^4^ | 1.14x10^4^ | 8.71x10^3^ | 2.08x10^4^±7.1x10^3^ |
| **β-** | 1.31x10^5^ | 3.88x10^5^ | 3.38x10^4^ | 3.56x10^4^ | 8.79x10^4^ | 1.35x10^5^±6.6x10^4^ |
| **γ-** | 6.88x10^4^ | 7.95x10^4^ | 1.42x10^4^ | 7.36x10^3^ | 1.56x10^4^ | 3.71x10^4^±1.5x10^4^ |
| **δ-** | 0.00 | 5.85x10^4^ | 6.50x10^3^ | 1.02x10^3^ | 0.00 | 1.32x10^4^±1.1x10^4^ |
| **ε-** | 1.45x10^4^ | 1.03x10^4^ | 6.19x10^2^ | 5.69x10^3^ | 0.00 | 6.21x10^3^±2.8x10^3^ |
| **Pla** | 0.00 | 4.17x10^4^ | 1.05x10^3^ | 4.42x10^3^ | 4.97x10^3^ | 1.04x10^4^±7.9x10^3^ |
| **CF** | 2.97x10^3^ | 1.03x10^5^ | 1.39x10^4^ | 3.14x10^4^ | 1.54x10^4^ | 3.34x10^4^±1.8x10^4^ |
|  | **Point 3 – Industrial/Urban** | | | | |  |
|  | **Oct-13** | **Mar-14** | **Oct-14** | **Apr-15** | **Oct-15** | **Average** |
| **α-** | 2.51x10^5^ | 4.48x10^3^ | 2.84x10^4^ | 4.91x10^4^ | 4.31x10^5^ | 1.53x10^5^±8.2x10^4^ |
| **β-** | 4.17x10^5^ | 9.35x10^4^ | 1.78x10^5^ | 6.72x10^4^ | 3.04x10^5^ | 2.12x10^5^±6.6x10^4^ |
| **γ-** | 2.74x10^5^ | 3.22x10^4^ | 1.54x10^4^ | 2.57x10^4^ | 1.40x10^5^ | 9.75x10^4^±5.0x10^4^ |
| **δ-** | 2.16x10^5^ | 1.42x10^4^ | 8.05x10^4^ | 1.86x10^3^ | 1.50x10^5^ | 9.26x10^4^±4.1x10^4^ |
| **ε-** | 5.98x10^4^ | 2.84x10^3^ | 6.07x10^3^ | 6.77x10^3^ | 1.32x10^4^ | 1.77x10^4^±1.1x10^4^ |
| **Pla** | 2.78x10^4^ | 0.00 | 3.59x10^3^ | 2.45x10^3^ | 1.30x10^5^ | 3.27x10^4^±2.5x10^4^ |
| **CF** | 2.73x10^5^ | 9.78x10^3^ | 2.39x10^5^ | 2.62x10^4^ | 3.52x10^5^ | 1.80x10^5^±6.9x10^4^ |
|  | **Point 4 - Urban** | | | | |  |
|  | **Oct-13** | **Mar-14** | **Oct-14** | **Apr-15** | **Oct-15** | **Average** |
| **α-** | 4.66x10^5^ | 4.47x10^3^ | 5.36x10^4^ | 4.60x10^3^ | 8.78x10^4^ | 1.23x10^5^±8.7x10^4^ |
| **β-** | 7.26x10^5^ | 3.05x10^5^ | 3.03x10^5^ | 4.89x10^4^ | 2.10x10^5^ | 3.19x10^5^±1.1x10^5^ |
| **γ-** | 2.04x10^5^ | 4.39x10^4^ | 1.86x10^4^ | 9.65x10^3^ | 6.39x10^4^ | 6.80x10^4^±3.5x10^4^ |
| **δ-** | 1.39x10^5^ | 8.23x10^3^ | 2.31x10^4^ | 2.63x10^3^ | 0.00 | 3.45x10^4^±2.6x10^4^ |
| **ε-** | 3.50x10^4^ | 8.69x10^3^ | 0.00 | 5.92x10^3^ | 0.00 | 9.91x10^3^±6.5x10^3^ |
| **Pla** | 5.90x10^4^ | 0.00 | 0.00 | 3.97x10^3^ | 0.00 | 1.26x10^4^±1.2x10^4^ |
| **CF** | 5.66x10^4^ | 3.59x10^4^ | 7.81x10^4^ | 6.38x10^3^ | 1.17x10^5^ | 5.88x10^4^±1.9x10^4^ |

^1^: Sample from Oct-13 excluded (see text).

**Table SI9: Spearman’s r correlation statistics. Correlation between microbiological parameters (Bacterial phylogenetic results) and grouped chemical compounds by including all samplings in the correlations and evaluating sites separately. Correlation values (r), corrected for multiple testing (Bonferroni), were considered significant if P < 0.001. Values in bold highlight significant correlations. α-, β-, γ-, δ-, ε-: *Proteobacteria*; Pla: *Planctomycetes*; CF: *Cytophaga-Flavobacterium lineage of the Bacteroidetes*.**

| **Point 1 - Pristine** | | | | |  | |  | |  | |  | |  | |
| --- | --- | --- | --- | --- | --- | --- | --- | --- | --- | --- | --- | --- | --- | --- |
|  | **PAHs** | | **Benzotriazoles** | | **Pharmaceuticals** | | **Pesticides** | | **Perfluorinated compounds** | | **Ions and nutrients** | | **Metals** | |
|  | r | P | r | P | r | P | r | P | r | P | r | P | r | P |
| **α-** | 0.4890 | 0.0899 | 0.3791 | 0.2014 | 0.6464 | 0.0170 | 0.3549 | 0.2341 | -0.3356 | 0.2623 | 0.6374 | 0.0191 | 0.5934 | 0.0325 |
| **β-** | 0.3736 | 0.2086 | 0.6539 | 0.0153 | 0.6243 | 0.0226 | 0.5255 | 0.0652 | 0.2944 | 0.3289 | 0.5714 | 0.0413 | 0.6593 | 0.0142 |
| **γ-** | 0.2363 | 0.4371 | 0.4286 | 0.1440 | 0.4033 | 0.1718 | 0.3466 | 0.2459 | 0.2503 | 0.4094 | 0.3517 | 0.2387 | 0.4835 | 0.0941 |
| **δ-** | 0.0000 | 1.0000 | 0.3571 | 0.2309 | 0.3978 | 0.1783 | 0.2806 | 0.3531 | 0.2751 | 0.3630 | 0.2473 | 0.4154 | 0.4231 | 0.1498 |
| **ε-** | 0.4819 | 0.0954 | 0.5154 | 0.0715 | 0.4846 | 0.0933 | 0.2664 | 0.3790 | 0.0530 | 0.8635 | 0.4095 | 0.1647 | 0.5794 | 0.0379 |
| **Pla** | 0.1768 | 0.5634 | -0.2763 | 0.3609 | -0.1278 | 0.6774 | -0.2545 | 0.4014 | -0.3347 | 0.2636 | -0.0994 | 0.7465 | -0.1271 | 0.6791 |
| **CF** | 0.4121 | 0.1618 | 0.5714 | 0.0413 | 0.6740 | 0.0115 | 0.6327 | 0.0203 | 0.2228 | 0.4643 | 0.5275 | 0.0640 | 0.6758 | 0.0112 |
| **Point 2 - Agricultural** | | |  |  |  |  |  |  |  |  |  |  |  |  |
|  | r | P | r | P | r | P | r | P | r | P | r | P | r | P |
| **α-** | 0.4559 | 0.0659 | 0.4853 | 0.0483 | 0.6757 | 0.0029 | 0.4942 | 0.0438 | -0.0711 | 0.7862 | 0.6128 | 0.0089 | 0.6226 | 0.0076 |
| **β-** | 0.3946 | 0.1170 | 0.6936 | 0.0020 | 0.6757 | 0.0029 | 0.6168 | 0.0084 | 0.4427 | 0.0752 | 0.5539 | 0.0211 | 0.6642 | 0.0036 |
| **γ-** | 0.2745 | 0.2863 | 0.4951 | 0.0433 | 0.4742 | 0.0545 | 0.5064 | 0.0380 | 0.4022 | 0.1095 | 0.3652 | 0.1495 | 0.5221 | 0.0316 |
| **δ-** | 0.1745 | 0.5031 | 0.3882 | 0.1236 | 0.4680 | 0.0582 | 0.1696 | 0.5151 | 0.1770 | 0.4967 | 0.3759 | 0.1370 | 0.5700 | 0.0169 |
| **ε-** | 0.4070 | 0.1050 | 0.5683 | 0.0173 | 0.5000 | 0.0410 | 0.4569 | 0.0652 | 0.1924 | 0.4593 | 0.4045 | 0.1073 | 0.6130 | 0.0089 |
| **Pla** | 0.3613 | 0.1542 | -0.0925 | 0.7241 | 0.1286 | 0.6229 | -0.0938 | 0.7204 | -0.0345 | 0.8953 | 0.0986 | 0.7064 | 0.0875 | 0.7383 |
| **CF** | 0.4706 | 0.0566 | 0.5172 | 0.0335 | 0.7175 | 0.0012 | 0.4831 | 0.0495 | 0.2538 | 0.3256 | 0.5858 | 0.0135 | 0.7181 | 0.0012 |

**Table SI9: Spearman’s r correlation statistics (continued).**

| **Point 3 – Industrial/Urban** | | | | | | |  | |  |  |  |  |  |  |  |  |  |
| --- | --- | --- | --- | --- | --- | --- | --- | --- | --- | --- | --- | --- | --- | --- | --- | --- | --- |
|  | | **PAHs** | | **Benzotriazoles** | | | | **Pharmaceuticals** | | **Pesticides** | | **Perfluorinated compounds** | | **Ions and nutrients** | | **Metals** | |
|  | r | | P | | r | P | r | | P | r | P | r | P | r | P | r | P |
| **α-** | 0.4755 | | 0.0537 | | 0.5270 | 0.0297 | **0.7469** | | **0.0006** | 0.5187 | 0.0329 | 0.0625 | 0.8115 | 0.6618 | 0.0038 | 0.6814 | 0.0026 |
| **β-** | 0.4044 | | 0.1074 | | 0.6985 | 0.0018 | 0.6708 | | 0.0032 | 0.6364 | 0.0060 | 0.5064 | 0.0380 | 0.5735 | 0.0161 | 0.6569 | 0.0042 |
| **γ-** | 0.3382 | | 0.1842 | | 0.5686 | 0.0172 | 0.5725 | | 0.0163 | 0.4966 | 0.0426 | 0.4856 | 0.0482 | 0.4657 | 0.0596 | 0.5907 | 0.0125 |
| **δ-** | 0.1275 | | 0.6257 | | 0.5322 | 0.0279 | 0.5741 | | 0.0160 | 0.3914 | 0.1203 | 0.3472 | 0.1721 | 0.3899 | 0.1218 | 0.5923 | 0.0122 |
| **ε-** | 0.4740 | | 0.0546 | | 0.6179 | 0.0082 | 0.6045 | | 0.0102 | 0.4196 | 0.0936 | 0.2918 | 0.2558 | 0.5038 | 0.0392 | 0.6998 | 0.0018 |
| **Pla** | 0.3710 | | 0.1426 | | 0.1106 | 0.6727 | 0.2857 | | 0.2663 | 0.1303 | 0.6182 | 0.1266 | 0.6282 | 0.1401 | 0.5919 | 0.1843 | 0.4789 |
| **CF** | 0.3775 | | 0.1353 | | 0.6299 | 0.0067 | **0.7764** | | **0.0002** | 0.6352 | 0.0061 | 0.3949 | 0.1168 | 0.5490 | 0.0225 | **0.7279** | **0.0009** |
| **Point 4 - Urban** | | | | |  |  |  | |  |  |  |  |  |  |  |  |  |
|  | r | | P | | r | P | r | | P | r | P | r | P | r | P | r | P |
| **α-** | 0.4755 | | 0.0537 | | 0.5784 | 0.0150 | **0.7543** | | **0.0005** | 0.4574 | 0.0649 | -0.0478 | 0.8554 | 0.6912 | 0.0021 | 0.6814 | 0.0026 |
| **β-** | 0.3309 | | 0.1945 | | 0.7034 | 0.0016 | 0.6364 | | 0.0060 | 0.6021 | 0.0105 | 0.4329 | 0.0826 | 0.5613 | 0.0191 | 0.6128 | 0.0089 |
| **γ-** | 0.2966 | | 0.2477 | | 0.6177 | 0.0082 | 0.5627 | | 0.0187 | 0.4329 | 0.0826 | 0.3581 | 0.1582 | 0.4779 | 0.0523 | 0.5637 | 0.0184 |
| **δ-** | -0.1057 | | 0.6865 | | 0.3047 | 0.2344 | 0.3153 | | 0.2177 | 0.3467 | 0.1728 | 0.2975 | 0.2462 | 0.1548 | 0.5531 | 0.3710 | 0.1426 |
| **ε-** | 0.2831 | | 0.2709 | | 0.4434 | 0.0746 | 0.3893 | | 0.1225 | 0.3666 | 0.1478 | 0.2068 | 0.4258 | 0.3031 | 0.2369 | 0.5173 | 0.0334 |
| **Pla** | 0.1825 | | 0.4833 | | -0.1023 | 0.6959 | 0.0581 | | 0.8247 | 0.0666 | 0.7994 | 0.0864 | 0.7417 | -0.0691 | 0.7923 | -0.0247 | 0.9252 |
| **CF** | 0.3628 | | 0.1524 | | 0.6740 | 0.0030 | **0.7764** | | **0.0002** | 0.5714 | 0.0166 | 0.2771 | 0.2815 | 0.5882 | 0.0130 | **0.7328** | **0.0008** |

### References

Costanzo, S.D., Watkinson, A.J., Murby, E.J., Kolpin, D.W., Sandstrom, M.W. 2007. Is there a risk associated with the insect repellent DEET (N, N-diethyl-m-toluamide) commonly found in aquatic environments? Science of the Total Environment 384, 214–220.

De Laurentiis, E., Chiron, S., Kouras-Hadef, S., Richard, C., Minella, M., Maurino, V., Minero, C., Vione, D. 2012. Photochemical fate of carbamazepine in surface freshwaters: Laboratory measures and modeling. Environmental Science & Technology 46, 8164−8173.

European Union, 2013. Directive 2008/105/EC of the European Parliament and of the Council of 16 December 2008 on environmental quality standards in the field of water policy, as amended by Directive 2013/39/EU of the European Parliament and of the Council of 12 August 2013.

Fenet, H., Mathieu, O., Mahjoub, O., Li, Z., Hillaire-Buys, D., Casellas, C., Gomez, E. 2012. Carbamazepine, carbamazepine epoxide and dihydroxycarbamazepine sorption to soil and occurrence in a wastewater reuse site in Tunisia. Chemosphere 88, 49–54.

Hummel, D., Löffler, D., Fink, G., Ternes, T.A. 2006. Simultaneous determination of psychoactive drugs and their metabolites in aqueous matrices by liquid chromatography mass spectrometry. Environ. Sci. Technol. 40, 7321–7328.

Johnson, A.C., Keller, V., Dumont, E., Sumpter, J.P. 2015. Assessing the concentrations and risks of toxicity from the antibiotics ciprofloxacin, sulfamethoxazole, trimethoprim and erythromycin in European rivers. Science of the Total Environment 511, 747–755.

Loos, R., Locoro, G., Contini, S. 2010. Occurrence of polar organic contaminants in the dissolved water phase of the Danube River and its major tributaries using SPE-LC-MS2 analysis. Water Research 44, 2325-2335.

Miao, X.-S., Metcalfe, C.D. 2003. Determination of carbamazepine and its metabolites in aqueous samples using liquid chromatography–electrospray tandem mass spectrometry. Analytical Chemistry 75, 3731–3738.

Patrolecco, L., Rauseo, J., Ademollo, N., Grenni, P., Cardoni, M., Levantesi, C., Luprano, M.L., Barra Caracciolo A. 2018 Persistence of the antibiotic sulfamethoxazole in river water alone or in the co-presence of ciprofloxacin. Science of the Total Environment 640-641,1438-1446.

Schreiner, V.C., Szöcs, E., Bhowmik, A.K., Vijver, M.G., Schäfer, R.B. 2016. Pesticide mixtures in streams of several European countries and the USA. Science of the Total Environment 573, 680–689.

Straub, J.O. 2016. Aquatic enviornmental risk assessment for human use of the old antibiotic sulfamethoxazole in Europe. Environmental Toxicology and Chemistry 35 (4), 767-779.

Tiedeken, E.J., Tahar, A., McHugh, B., Rowan, N.J., 2017. Monitoring, sources, receptors, and control measures for three European Union watch list substances of emerging concern in receiving waters – A 20year systematic review. Sci. Total Environ. 574, 1140–1163.

Valsecchi, S., Rusconi, M., Mazzoni, M., Viviano, G., Pagnotta, R., Zaghi, C., Serrini, G., Polesello, S. 2015. Occurrence and sources of perfluoroalkyl acids in Italian river basins. Chemosphere 129, 126–134.

Weiss, S., Jakobs, J., Reemtsma, T., 2006. Discharge of three benzotriazole corrosion inhibitors with municipal wastewater and improvements by membrane bioreactor treatment and ozonation. Environmental Science & Technology 40, 7193–7199.
